# Homology-mediated end joining-based targeted integration using CRISPR/Cas9

**DOI:** 10.1038/cr.2017.76

**Published:** 2017-05-19

**Authors:** Xuan Yao, Xing Wang, Xinde Hu, Zhen Liu, Junlai Liu, Haibo Zhou, Xiaowen Shen, Yu Wei, Zijian Huang, Wenqin Ying, Yan Wang, Yan-Hong Nie, Chen-Chen Zhang, Sanlan Li, Leping Cheng, Qifang Wang, Yan Wu, Pengyu Huang, Qiang Sun, Linyu Shi, Hui Yang

**Affiliations:** 1 Institute of Neuroscience, State Key Laboratory of Neuroscience, Key Laboratory of Primate Neurobiology, CAS Center for Excellence in Brain Science and Intelligence Technology, Shanghai Institutes for Biological Sciences, Chinese Academy of Sciences, Shanghai 200031, China; 2 College of Life Sciences, University of Chinese Academy of Sciences, Beijing 100049, China; 3 School of Life Science and Technology, ShanghaiTech University, Shanghai 201210, China; 4 Institute of Biochemistry and Cell Biology, Shanghai Institutes for Biological Sciences, Chinese Academy of Sciences, Shanghai 200031, China; 5 Shanghai University, Shanghai 200444, China; 6 National Institute of Biological Sciences, Beijing 102206, China

**Keywords:** homology-mediated end joining, CRISPR/Cas9, monkey embryos, neurons, knock-in

## Abstract

Targeted integration of transgenes can be achieved by strategies based on homologous recombination (HR), microhomology-mediated end joining (MMEJ) or non-homologous end joining (NHEJ). The more generally used HR is inefficient for achieving gene integration in animal embryos and tissues, because it occurs only during cell division, although MMEJ and NHEJ can elevate the efficiency in some systems. Here we devise a homology-mediated end joining (HMEJ)-based strategy, using CRISPR/Cas9-mediated cleavage of both transgene donor vector that contains guide RNA target sites and ∼800 bp of homology arms, and the targeted genome. We found no significant improvement of the targeting efficiency by the HMEJ-based method in either mouse embryonic stem cells or the neuroblastoma cell line, N2a, compared to the HR-based method. However, the HMEJ-based method yielded a higher knock-in efficiency in HEK293T cells, primary astrocytes and neurons. More importantly, this approach achieved transgene integration in mouse and monkey embryos, as well as in hepatocytes and neurons *in vivo*, with an efficiency much greater than HR-, NHEJ- and MMEJ-based strategies. Thus, the HMEJ-based strategy may be useful for a variety of applications, including gene editing to generate animal models and for targeted gene therapies.

## Introduction

Targeted integration of transgenes is usually achieved by a homologous recombination (HR)-mediated method^1,2^. It requires a repair template that harbors left and right homology arms (HAs) (500-3 000 bp), thus allowing precise insertion of large DNA fragments. Custom-designed nucleases, including zinc-finger nucleases^3,4,5,6^, transcription activator-like effector nucleases^2,7^ and the clustered regularly interspaced short palindromic repeats (CRISPR)/CRISPR-associated protein-9 nuclease (Cas9) system^1,8,9^, greatly facilitate targeted integration of transgenes by generating a targeted DNA double-strand break (DSB) in the genome. Once a DSB is created, externally supplied DNA fragments can be introduced around the cleavage site during its repair by HR. However, this approach is generally inefficient in animal embryos and tissues *in vivo*^10,11^, because HR is active only during the late S/G2 phase.

Recently, non-homologous end joining (NHEJ) or microhomology-mediated end joining (MMEJ)-based methods capable of integrating long exogenous DNA fragments into the genome at relatively high frequencies were reported^12,13,14,15,16,17,18,19^. In these methods, a targeted genomic locus and a donor vector with no HA or with microhomology arms (5-25 bp) are simultaneously cleaved by programmable nucleases and then connected to each other through NHEJ or MMEJ, resulting in targeted transgene integration^12,14^. However, NHEJ-based targeted integration introduced random directions in integration and various types of indels at the junctions, making it difficult to construct endogenous and exogenous in-frame fusion genes for chimeric protein production^12,13,17^. MMEJ-based targeted integration exhibited low efficiency in cultured cells^14,20^.

**Figure 1:**
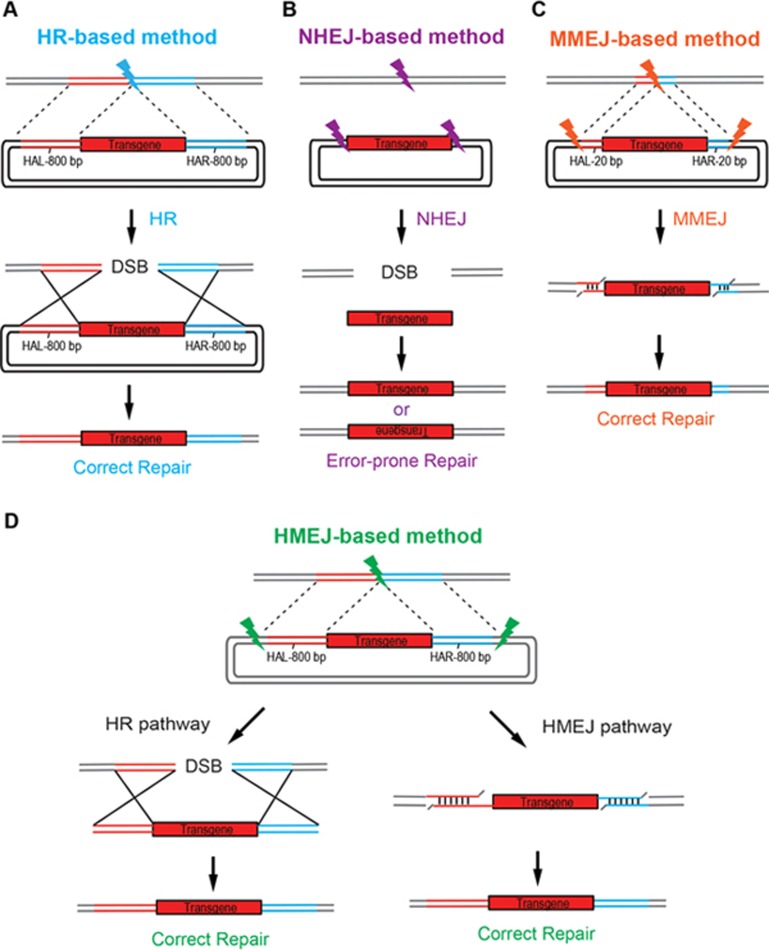
Schematic overview of HR-, NHEJ-, MMEJ- and HMEJ-mediated gene knock-in. **(A)** The HR-based method requires relatively long homology arms (800 bp). **(B)** The NHEJ-based method requires sgRNA target sites but without any homology arms. NHEJ repair system introduced various types of indel mutations at the junctions. **(C)** The MMEJ-based method requires sgRNA target sites as well as short homology arms (5-20 bp). **(D)** The HMEJ-based method requires sgRNA target sites as well as long homology arms (800 bp). HR and HMEJ mechanisms may be involved in this method. HAL/HAR, left/right homology arm.

In this study, we have examined the possibility that CRISPR/Cas9-mediated DNA cleavage on an HR donor could improve the efficiency of homology-mediated gene integration especially in non-dividing cells. We surmised that targeted integration by this strategy could be achieved via the HR pathway as well as through a new DSB repair pathway requiring homology-mediated end joining (HMEJ), thus improving knock-in efficiency (Figure 1). Compared with MMEJ, HMEJ harbors longer and perhaps more stable HAs to achieve targeted integration with higher efficiency. For this purpose, we devise a new strategy for precise integration, based on HMEJ, using a donor with single guide RNA (sgRNA) target sites and long HAs (∼800 bp). We found that the HMEJ-based strategy showed a higher knock-in efficiency than all existing strategies in many systems, including cultured cells, animal embryos and tissues *in vivo*.

## Results

### *In vitro* genome editing using the HMEJ-based method

**Figure 2:**
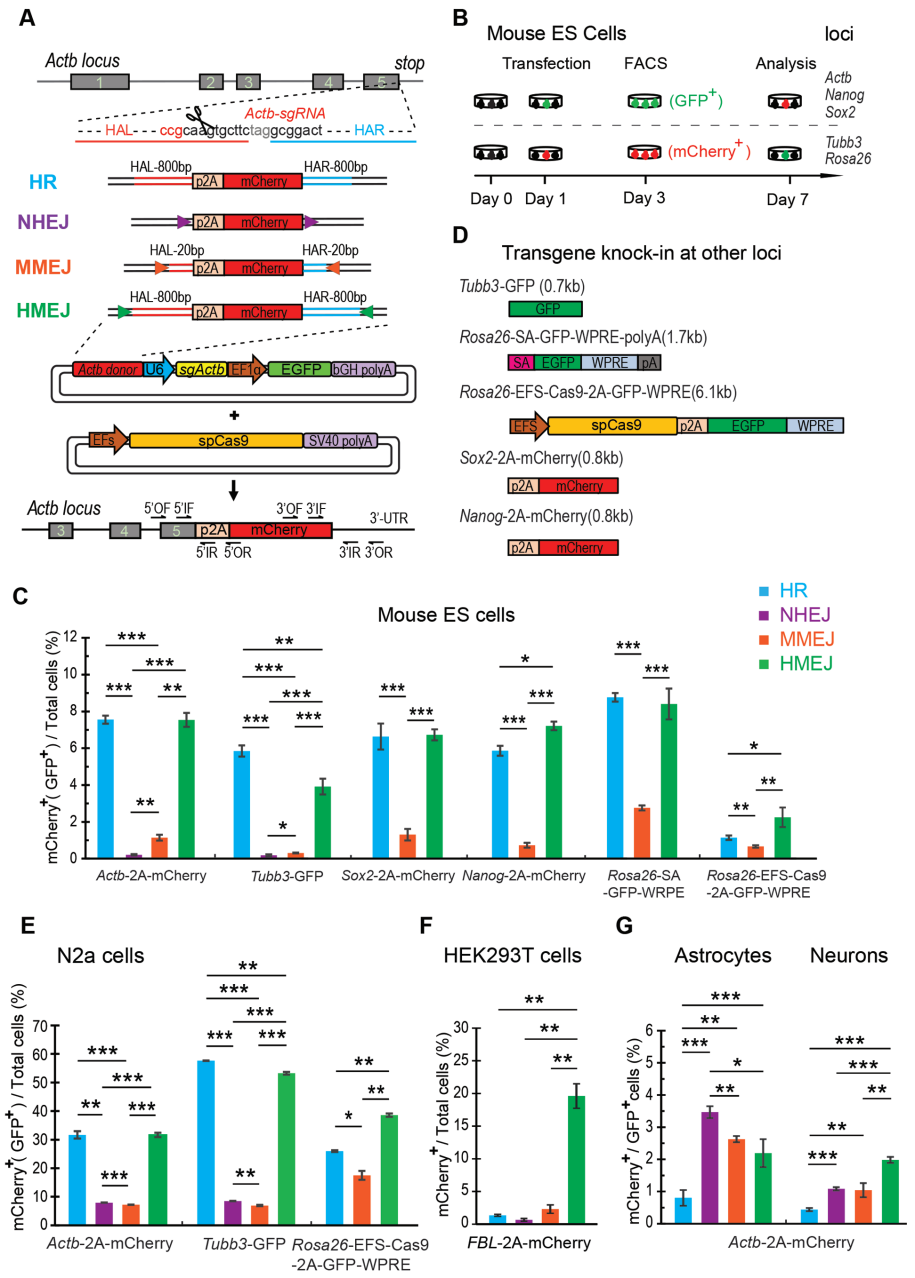
*In vitro* genome editing via HMEJ-mediated targeted integration **(A)** Schematic overview of four gene targeting strategies at the *Actb* locus. HAL/HAR, left/right homology arm; triangles, sgRNA target sites; OF/OR, outer forward/reverse primer; IF/IR, inner forward/reverse primer. **(B)** Experimental scheme for targeted *Actb*-2A-mCherry knock-in in mouse ES cells. Cells were transfected with donors/sgRNA/GFP or donors/sgRNA/mCherry and Cas9, and transfected cells were sorted based on GFP or mCherry signals 2 days after transfections. Knock-in efficiencies were evaluated by FACS based on the ratio of GFP^+^ or mCherry^+^ cells among total transfected cells 4 days after the first sorting. **(C)** Relative knock-in efficiency of HR-, NHEJ-, MMEJ- and HMEJ-based strategies in mouse ES cells at various loci measured by the percentage of mCherry^+^ (or GFP^+^) cells among total transfected cells. Note that the NHEJ-based method was not performed at *Sox2*, *Nanog* and *Rosa26* loci. **(D)** Schematic overview of insertion fragments at different loci. **(E**, **F)** Relative knock-in efficiency of HR-, NHEJ-, MMEJ- and HMEJ-based strategies in N2a cells **(E)** and HEK293T cells **(F)** at various loci measured by the percentage of mCherry^+^ (or GFP^+^) cells among total transfected cells. Note that the NHEJ-based method was not performed at the *Rosa26* locus. **(G)** Relative knock-in efficiency in primary astrocytes and neurons measured by the percentage of mCherry^+^ cells among GFP^+^ cells. The results in panels **C**, **E**, **F** and **G** were presented as mean ± SD. ^*^P < 0.05, ^**^P < 0.01, ^***^P < 0.001, unpaired Student's *t*-test.

We first examined whether the HMEJ-based method showed a more robust knock-in *in vitro* compared with HR-, NHEJ- and MMEJ-based methods using CRISPR/Cas9. To test this idea, we compared the knock-in efficiency using four types of donors: an HMEJ donor (sgRNA target sites plus long HAs (800 bp)), an HR donor (only long HAs), an NHEJ donor (only sgRNA target sites) and an MMEJ donor (sgRNA target sites plus short HAs (20 bp)) (Figure 1). To evaluate knock-in efficiencies, we aimed to fuse a p2A-mCherry reporter gene to the last codon of the *Actb* gene in mouse embryonic stem (ES) cells. The resulting knock-in efficiencies are presented as percentages of mCherry^+^ cells (Figure 2A and 2B). At 7 days after transfecting mouse ES cells with donor/sgRNA plasmids and Cas9, the knock-in efficiency of the HMEJ-based method (7.54% ± 0.37%) was similar to the HR-based method (7.55% ± 0.22%), but higher than the MMEJ-based method (1.14% ± 0.16%) and the NHEJ-based method (0.21% ± 0.04%) (Figure 2C). Genotyping showed that HMEJ- and HR-mediated gene knock-in represented precise in-frame integrations at 5′ and 3′ junctions (Supplementary information, Figure S1).

We next examined knock-in efficiencies at other loci (*Tubb3*, *Rosa26*, *Sox2* and *Nanog*), with different insertion fragments (ranging in size from 0.7 kb to 6.1 kb), and observed similar trends (Figure 2C and 2D; Supplementary information, Figure S2). In addition, we tested the HMEJ-based method in N2a cells (a mouse neuroblastoma cell line) at the *Actb*, *Tubb3* and *Rosa26* loci, and also observed that HR- and HMEJ-based methods showed higher knock-in efficiency than NHEJ- and MMEJ-based methods (Figure 2E). Furthermore, we fused p2A-mCherry to the last exon of the human *fibrillarin* (*FBL*) gene in HEK293T cells (Supplementary information, Figure S2B). We found that the HMEJ-based method exhibited a much higher knock-in efficiency than the three other methods in HEK293T cells (Figure 2F), consistent with a recent study^20^.

To test whether HA length could affect the knock-in efficiency of the HMEJ-based method, we designed a series of HMEJ donors for p2A-mCherry knock-in at the *Actb* locus in mouse ES cells and N2a cells, with HA length in the range of 200-1 600 bp. We found that HAs of 800 bp and 1 600 bp showed a higher knock-in efficiency than HAs of 200 and 400 bp (Supplementary information, Figure S3). Due to the size limitation for *in vivo* application and plasmid construction, we used an HMEJ donor with HAs of 800 bp in the following experiments.

We also compared relative knock-in efficiencies at the *Actb* locus in primary astrocytes and neurons with the four types of donors described above (Supplementary information, Figure S4A and S4B). Five days after transfection via lentivirus, we measured the percentage of mCherry^+^ cells among GFP^+^ cells enriched by fluorescence-activated cell sorting (FACS) and found very few cells exhibited knock-ins with an HR donor (Figure 2G). By contrast, three other methods that used donor containing sgRNA target sites produced efficient mCherry knock-in in primary astrocytes and neurons (Figure 2G). Genotyping confirmed the precise integration in neurons mediated by the HMEJ-based method (Supplementary information, Figure S4C).

Together, these results indicated that the HMEJ-based method showed a similar transgene knock-in efficiency in mouse ES cells and N2a cells, but yielded a higher knock-in efficiency in HEK293T cells, primary astrocytes and neurons, compared with the HR-based method.

### Genome editing in mouse and monkey embryos using the HMEJ-based method

**Figure 3:**
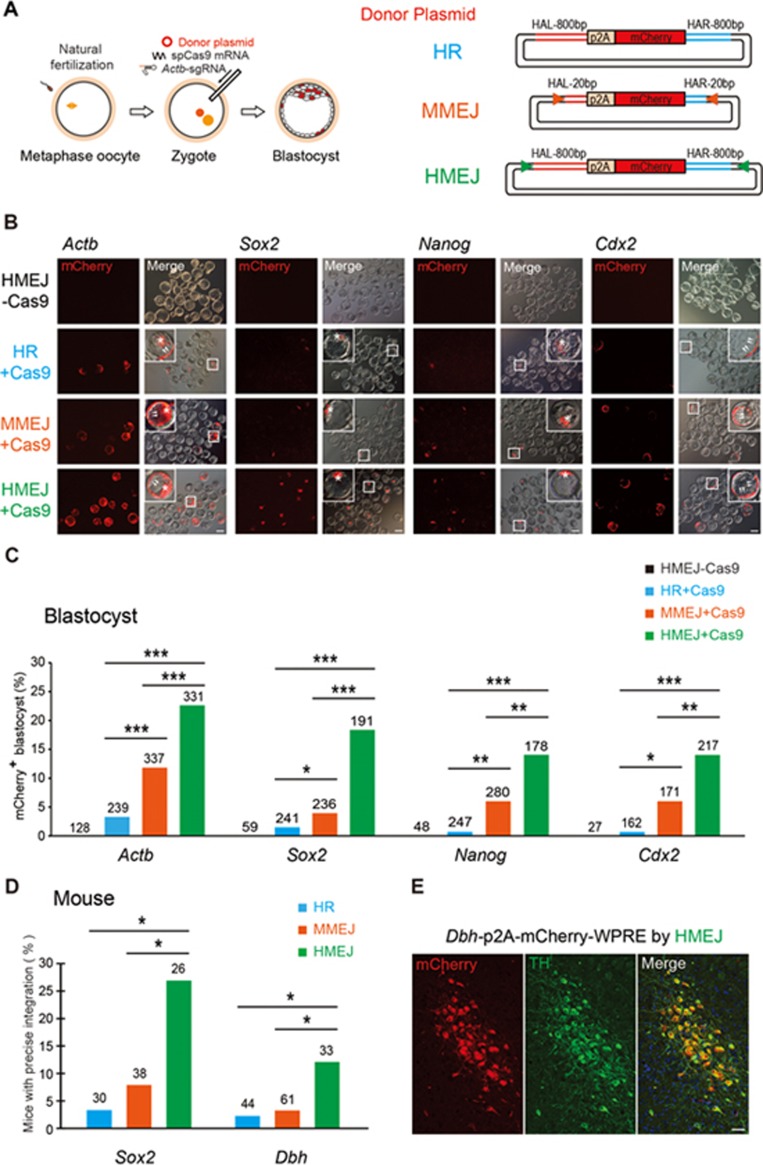
HMEJ-mediated targeted integration in mouse embryos. **(A)** Experimental design. Cas9 mRNA, sgRNA and donor vector were injected into mouse zygotes and the injected zygotes were cultured to the blastocyst stage to observe fluorescence and for genotyping analysis. **(B)** Representative immunofluorescence images of gene-edited mouse embryos at the blastocyst stage. Cas9 mRNA, sgRNA and each donor vector (HR, MMEJ or HMEJ) were injected into mouse zygotes and the injected zygotes were cultured to the blastocyst stage for fluorescence observation. The control, HMEJ donor without Cas9. Insets, higher magnification images. Scale bar, 50 μm. **(C)** Knock-in efficiencies indicated by percentage of mCherry^+^ blastocysts. Number above each bar, total blastocysts counted. **(D)** Efficiencies of mice with p2A-mCherry precise integration at *Sox2* and *Dbh* loci. Number above each bar, total mice counted. **C** and **D**, ^*^P < 0.05, ^**^P < 0.01, ^***^P < 0.001, *χ*^2^-test. **(E)** Representative immunofluorescence images of brain in 6-week-old mice with *Dbh*-p2A-mCherry knock-in by HMEJ-based method. Scale bar, 50 μm. Arrowheads, TE; Asterisk, ICM; TH, tyrosine hydroxylase.

To investigate whether the HMEJ strategy could improve knock-in efficiency in generating gene-modified mice, we injected Cas9 mRNA, sgRNA targeting the *Actb* gene and the HMEJ donor into mouse zygotes (Figure 3A). The injected zygotes were cultured into blastocysts and knock-in efficiencies were evaluated by mCherry fluorescence signals in blastocysts. Interestingly, we observed a much higher rate of mCherry^+^ blastocysts with the HMEJ donor (22.7%) than with the MMEJ donor (11.9%), HR donor (3.3%) or NHEJ donor (1.4%) (Figure 3B and 3C; Supplementary information, Figure S5A-S5C). Furthermore, the genotyping of individual mCherry^+^ blastocysts with knock-in at *Actb* by the HMEJ- or MMEJ-based methods showed that all examined integration events were precise in-frame integrations at 5′ and 3′ junctions (Supplementary information, Figure S5D and S5E). By contrast, the NHEJ-based method showed a low efficiency of gene knock-in and introduced indels at the junctions, consistent with previous reports^12,13^ (Supplementary information, Figure S5F). We also examined HMEJ-mediated knock-in efficiencies at other loci, including *Nanog* (pluripotency marker), *Sox2* (pluripotency marker) and *Cdx2* (trophectoderm (TE) marker), by fusing p2A-mCherry reporter to the last codon of the targeted genes. We found that HMEJ-based method exhibited the highest knock-in efficiencies at all three loci (Figure 3B and 3C). Notably, for mCherry^+^ blastocysts, mCherry was strictly expressed in the inner cell mass (ICM) for *Nanog* and *Sox2* knock-ins, and in the TE for the *Cdx2* knock-in, expression patterns indicating correct integration (Figure 3B). By contrast, mCherry^+^ cells were observed in both ICM and TE of *Actb* knock-in blastocysts (Figure 3B). Genotyping of individual mCherry^+^ blastocyst confirmed precise integration at 5′ and 3′ junctions of *Nanog*, *Sox2* and *Cdx2* loci by the HMEJ-based method (Supplementary information, Figure S6).

We next targeted the dopamine beta-hydroxylase (*Dbh*) gene, a marker for neurons expressing tyrosine hydroxylase (TH) and *Sox2*, and fused p2A-mCherry to the last codon of these targeted genes, to evaluate the HMEJ-based method for generating gene-modified mice (Supplementary information, Figure S7A). After transplantation of gene-edited embryos into pseudo-pregnant mice, we achieved gene-edited mice with normal birth rate by the HMEJ-based method and successfully obtained knock-in mice at *Dbh* (12.1%) and *Sox2* (26.9%) loci, with higher knock-in efficiency than HR- and MMEJ-based methods (Figure 3D, Supplementary information, Figure S7B, and Table S1). The immunostaining of brain tissues of *Dbh* knock-in mice showed that mCherry was specifically expressed in TH^+^ neurons, indicating the correct integration had occurred (Figure 3E). Precise integration of transgenes in all of these knock-in mice (*Sox2* and *Dbh*) was further confirmed by genotyping and DNA sequencing (Supplementary information, Figure S7C and S7D). Together, these results indicate that the HMEJ-based method showed much higher DNA integration efficiency than the three other strategies in the generation of gene-modified mice.

**Figure 4:**
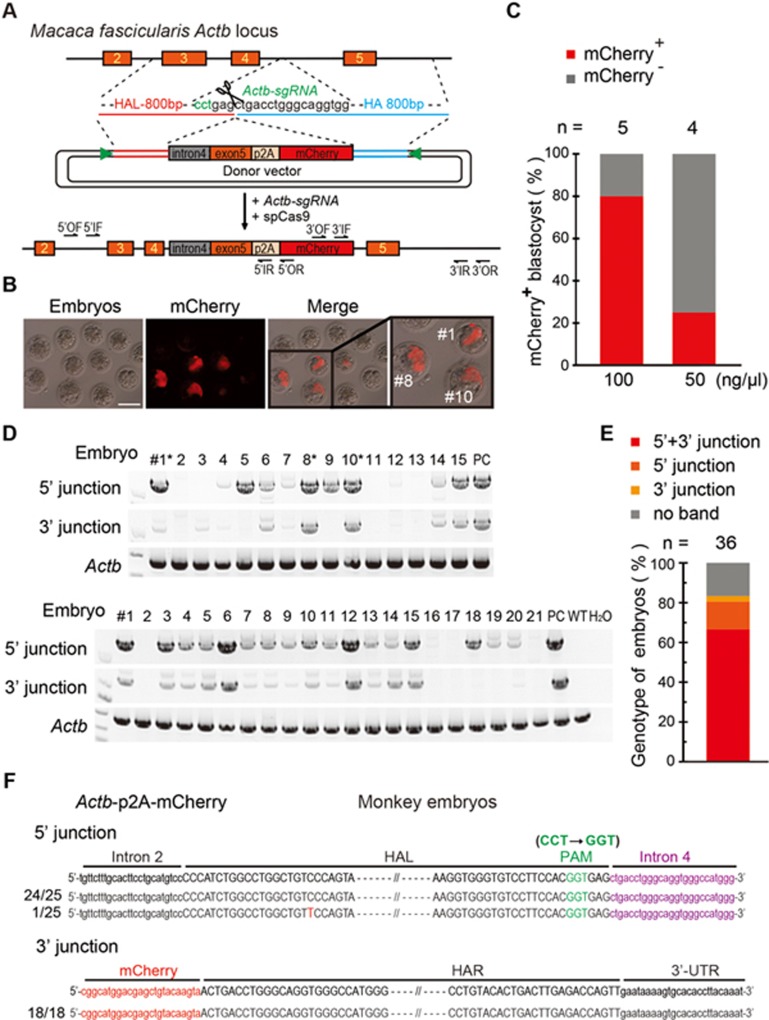
HMEJ-mediated targeted integration in monkey embryos. **(A)** Schematic overview of HMEJ-mediated gene targeting strategy at the *Actb* locus in monkey embryos. Intracytoplasmic sperm injection (ICSI) was performed on monkey oocyte and the Cas9 mix was injected 6 h later. The injected embryos were cultured for 7 days into blastocysts. **(B)** Representative immunofluorescence images of gene-edited monkey embryos at the blastocyst stage. Square, blastocysts shown at a higher resolution on the right panel. Scale bar, 100 μm. Numbered blastocysts were genotyped and labeled with ^*^ below. **(C)** Knock-in efficiencies with different concentrations of donor plasmids (100, 50 ng/μl) indicated by percentage of mCherry^+^ blastocysts. Number above the bar, total blastocysts counted. **(D)** Genotyping analysis of the injected embryos. PCR products amplified from 5′ and 3′ junction sites of DNA samples of individual monkey embryos on day 7 were sequenced and shown in **D**. PC, positive control from COS-7 cells with *Actb*-p2A-mCherry knock-in. ^*^, mcherry^+^ blastocyst shown in **B**. **(E)** Genotype of integration junctions in total injected embryos. Number above the bar, total embryos analysis. **(F)** Sequencing analysis of integration junctions of the injected embryos. PCR products amplified from the 5′ and 3′ junction sites of DNA samples extracted from individual embryos were sequenced. Number, total sample size. CCT to GGT, replace PAM sequence CCT of sgRNA to GGT to avoid recutting. Dashed lines mark the region omitted for clarity. Upper, homology arm; purple, intron 4; red, mCherry; green, PAM sequence. Dashed lines mark the region omitted for clarity.

Largely due to the low DNA cleavage efficiency of sgRNA in monkey embryos, no successful generation of a knock-in monkey has been reported^21,22,23,24^. We therefore tested whether the HMEJ-based method could efficiently generate a knock-in monkey. We aimed to insert *Actb*-intron 4-exon 5-2A-mCherry into intron 4 of the *Actb* locus to achieve mCherry expression under the control of the *Actb* promoter (Figure 4A). We first tested cleavage efficiency of sgRNAs (sgRNA-1 to -11) on monkey COS-7 cells (Supplementary information, Figure S8A and S8B). On the basis of T7E1 results, we co-injected Cas9 mRNA, sgRNA-5 with relatively higher cleavage activity (Supplementary information, Figure S8C and S8D), and the HMEJ donor into monkey embryos. At first, we injected the HMEJ donor at 100 ng/μl into 26 monkey embryos and obtained 4 mCherry^+^ blastocysts out of 5 blastocysts in total. Then, we injected the HMEJ donor at 50 ng/μl into 10 monkey embryos and obtained 1 mCherry^+^ blastocysts out of 4 blastocysts in total. Thus altogether, we obtained 5 mCherry^+^ blastocysts out of 9 (Figure 4B and 4C). We then amplified the integration junctions by PCR and found that among the 36 injected embryos, 29 were positive at the 5′ junction, 25 were positive at the 3′ junction and 24 were double positive at the 5′ and 3′ junctions (Figure 4D and 4E). The PCR products were directly sequenced and most of them (24 out of 25 PCR products at the 5′ junction and 18 out of 18 PCR products at the 3′ junction) were precise integrations (Figure 4F). Thus, HMEJ represents an efficient method for generating knock-in monkeys.

### *In vivo* genome editing using the HMEJ-based method

**Figure 5:**
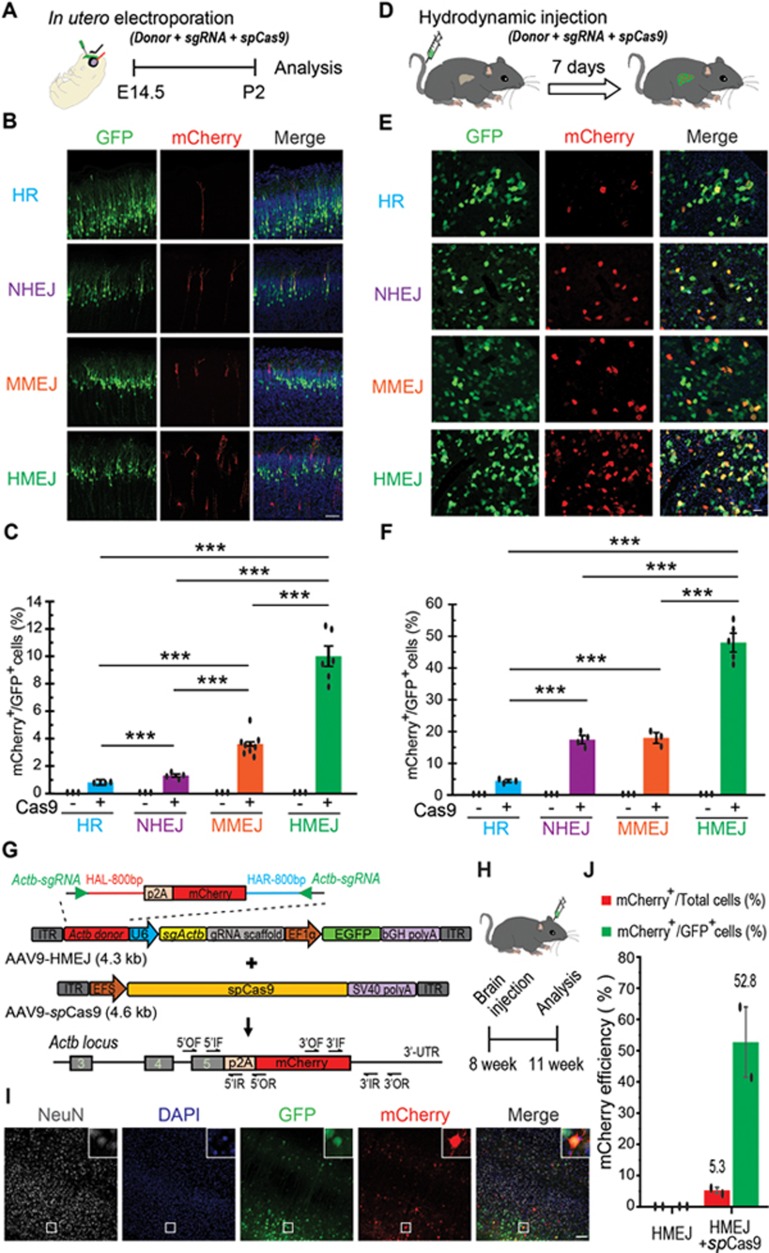
*In vivo* genome editing via HMEJ-mediated targeted integration. **(A)** Experimental scheme for targeted *Actb*-2A-mCherry knock-in in fetal brain via *in utero* electroporation. **(B)** Representative immunofluorescence images of neurons showing correct mCherry knock-in at the *Actb* locus with four gene targeting strategies. Scale bar, 100 μm. GFP, transfected cells. **(C)** Relative knock-in efficiency measured by the percentage of mCherry^+^ cells among GFP^+^ cells. **(D)** Experimental scheme for targeted *Actb*-2A-mCherry knock-in via hydrodynamic tail vein injection. **(E)** Representative immunofluorescence images of hepatocytes in liver sections at day 7 post injection. Scale bar, 50 μm. GFP, transfected cells. **(F)** Relative knock-in efficiency measured by the percentage of mCherry^+^ cells among GFP^+^ cells. Hepatocytes were harvested at day 7 post injection. **C** and **F**, results were obtained from at least three mice and presented as mean ± SD. The input data points were shown as black dots. ^***^P< 0.001, unpaired Student's *t*-test. **(G)** Schematic of HMEJ-AAV vectors for knock-in of p2A-mCherry to the last codon of the *Actb* gene. **(H)** Schematic of *in vivo* HMEJ-mediated knock-in via local AAV injections in adult mouse brain. **(I)** Representative immunofluorescence images of neurons in HMEJ-AAV-injected brain sections. Insets, higher magnification images. Scale bar, 100 μm. **(J)** Relative and absolute knock-in efficiencies measured by the percentage of mCherry^+^ cells among GFP^+^ cells or all DAPI^+^ cells, respectively. Results were obtained from two animals and presented as mean ± SD. At least 2 000 cells of each brain section and three brain sections of each animal were counted. The input data points were shown as black dots.

As previously reported, the widely used method of HR-based targeted genome editing is inefficient when applied to tissues *in vivo*^10^. We therefore set out to see if the HMEJ-based method could be applied for *in vivo* DNA integration. We first delivered *Actb*-HMEJ constructs to the E14.5 mouse brain using *in utero* electroporation (Figure 5A). Seven days after electroporation, brain sections were stained and counted. We observed that 10.0% ± 0.7% of electroporated cells (mCherry^+^/GFP^+^, relative efficiency) showed mCherry expression (Figure 5B and 5C). By contrast, only 0.8% ± 0.2%, 1.3% ± 0.1% and 3.6% ± 0.2% of electroporated cells were mCherry^+^ using HR, NHEJ and MMEJ donors, respectively (Figure 5B and 5C). We next delivered the HR, NHEJ, MMEJ and HMEJ constructs to mouse liver by hydrodynamic injection into the tail vein, and found that 4.5% ± 0.5%, 17.4% ± 1.3%, 18.0% ± 1.7% and 48.0% ± 2.9% of transfected hepatocytes, respectively, (mCherry^+^/GFP^+^, relative efficiency) showed mCherry expression at day 7 post injection (Figure 5D-5F). Precise integration by HMEJ in neurons and hepatocytes was confirmed by genotyping and DNA sequencing (Supplementary information, Figure S9).

For *in vivo* applications, we tested whether HMEJ-mediated targeted integration could be achieved by the delivery of Cas9 and the sgRNA/HMEJ donor using adeno-associated virus (AAV). Three weeks after injection of HMEJ-AAVs into the visual cortex (V1) of adult mice, brain sections were stained and counted (Figure 5G and 5H). In contrast to uninfected cells, 52.8% ± 11.3% of infected GFP^+^ cells were mCherry^+^ and most of them co-localized with NeuN (a neuron marker). This indicates that HMEJ-mediated targeted integration can be efficiently achieved in non-dividing cells (Figure 5I and 5J). Precise integration by HMEJ in neurons was further confirmed by genotyping and DNA sequencing (Supplementary information, Figure S9).

Together, our results indicate that the HMEJ-based method shows much higher DNA integration efficiency than HR-, NHEJ- and MMEJ-based methods *in vivo*.

### Mechanism of the HMEJ-based method

**Figure 6:**
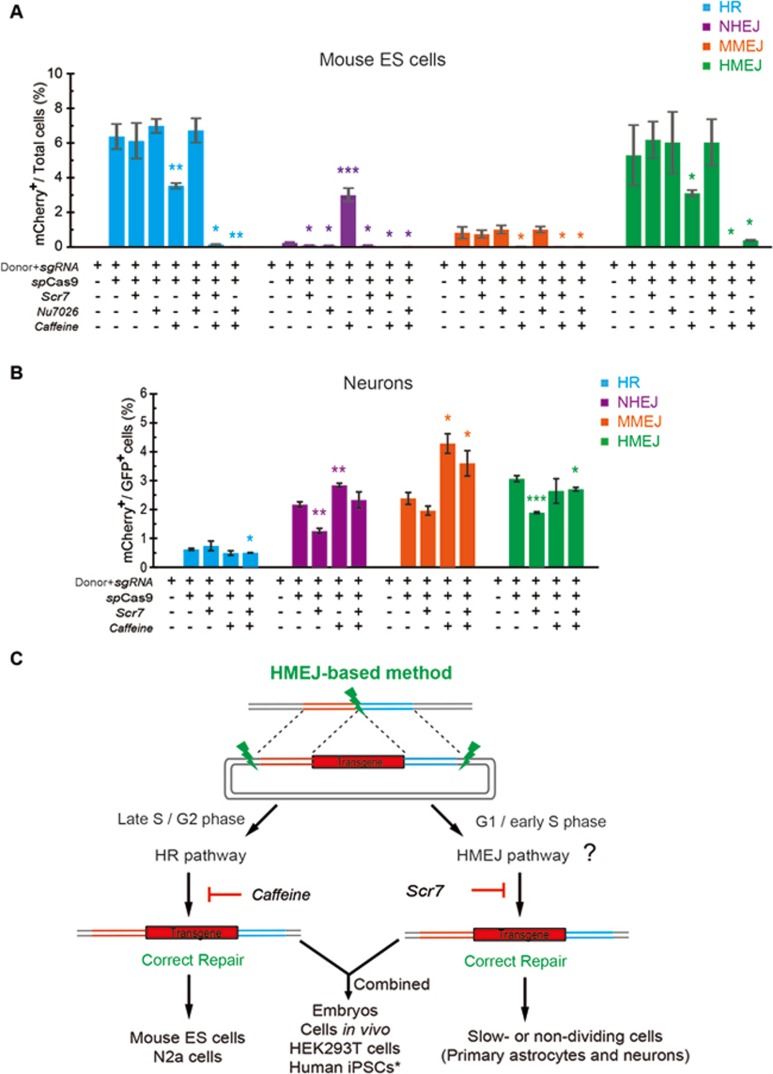
The effect of the NHEJ inhibitor and HR inhibitor on knock-in efficiencies by different strategies. **(A-B)** Knock-in efficiencies of mCherry knock-in at *Actb* locus by four strategies in mouse ES cells **(A)** and neurons **(B)** were measured by FACS and compared with the group treated with NHEJ inhibitor (Scr7 or Nu7026), HR inhibitor (caffeine) or both. Results were presented as mean ± SD. ^*^P < 0.05, ^**^P < 0.01, ^***^P < 0.001, unpaired Student's *t*-test. **(C)** Schematic overview of HMEJ-mediated gene knock-in in different types of cells. ^*^, reported in a recent report^20^.

Finally, we explored whether the HMEJ-based method depends on the HMEJ and HR pathways (Figure 1). Mouse ES cells and primary neurons were transfected with different donors for p2A-mCherry knock-in at the *Actb* locus and treated with the NHEJ inhibitor (Scr7 or Nu7026) and HR inhibitor (caffeine, a non-specific inhibitor of the ATM and ATR kinases involved in HR) during the transfection procedure (Figure 6A and 6B). Consistent with previous studies^12,17^, we found that the NHEJ inhibitor and HR inhibitor blocked and promoted NHEJ-mediated knock-in, respectively, in both mouse ES cells and neurons. Interestingly, the HR inhibitor blocked MMEJ-mediated knock-in in mouse ES cells but promoted MMEJ-mediated knock-in in neurons (Figure 6A and 6B). As to the HMEJ-mediated knock-in, we found that HR inhibitor significantly decreased HMEJ-mediated knock-in in mouse ES cells but had no effect on neurons. By contrast, the NHEJ inhibitor significantly decreased HMEJ-mediated knock-in in neurons but had no effect in mouse ES cells (Figure 6A and 6B). In mouse ES cells, the HMEJ-based method showed similar knock-in efficiencies with HR-based methods. In primary neurons, the HMEJ-based method showed similar knock-in efficiencies as with NHEJ and MMEJ-based methods (Figure 6A and 6B). These results suggest that HMEJ-based knock-in is mainly mediated by the HR pathway in mouse ES cells, and NHEJ inhibitor affected HMEJ pathway in primary neurons (Figure 6C). On the basis of the similar patterns of knock-in efficiency between mouse ES cells and N2a cells (Figure 2C and 2E), and between primary neurons and astrocytes (Figure 2G), we hypothesized that the HMEJ-based method may be mediated through the HR pathway in N2a cells and the HMEJ pathway in primary astrocytes (Figure 6C).

Concerning mouse embryos, HEK293T cells, cells *in vivo* and recently reported human-induced pluripotent stem cells (iPSCs)^20^, the HMEJ-based method exhibited much higher knock-in efficiencies than the three other methods. We suppose that insertion using the HMEJ-based method in these types of cells may be mediated by the HR pathway, as well as HMEJ pathway (Figure 6C). This may account for the high knock-in efficiency achieved by the HMEJ-based method.

Notably, we observed that NHEJ and HR inhibitors showed synergetic effects in inhibiting HR-based and HMEJ-based integration efficiency in mouse ES cells (Figure 6A). We think that the HR pathway is a dominant route for HR-based targeted integration. Thus, NHEJ inhibitors showed no obvious effect on HR efficiency and HR inhibitors blocked HR-based knock-in. However, when the HR pathway is blocked in mouse ES cells, the HR-based method may execute an alternative route to mediate transgene integration, possibly through the ligase IV-dependent pathway (inhibited by NHEJ inhibitors Scr7 or Nu7026), because we could still observe some knock-in events occurring. When NHEJ inhibitors and HR inhibitors are simultaneously added, HR-based integrations were completely blocked. The HMEJ-based method may operate through a similar pathway as the HR-based method in mouse ES cells. Thus, the HMEJ-based method showed a similar pattern in affecting HR and HMEJ efficiency.

## Discussion

Here we have described an HMEJ-based strategy that has shown the highest knock-in efficiencies among all existing strategies in many systems, including cultured cells, animal embryos and tissues *in vivo*. The robust DNA knock-in achieved by this method may be attributed to the use of HR pathway and HMEJ pathway simultaneously in the process of targeted transgene integration. Compared with the HR-based method, the HMEJ-based method showed a similar transgene knock-in efficiency in mouse ES cells and was also affected by the HR inhibitor. However, the HMEJ-based method was not affected by the HR inhibitor in primary neurons, and showed a much higher (6-15-folds) knock-in efficiency than the HR-based method in HEK293T cells, embryos and in slow or non-dividing cells such as primary astrocytes, neurons and hepatocytes. We hypothesize that the HMEJ-based method requires a new repair pathway, which we term the HMEJ pathway, to mediate highly efficient targeted integration in certain types of cells. First, HR only occurs during the late S/G2 phase, but the HMEJ-based method exhibits a high knock-in efficiency in non-dividing cells, including primary neurons and neurons in adult mice. Thus, the HMEJ-based method is not likely to use the HR pathway to mediate targeted integration of transgenes in neurons. Second, with the NHEJ or MMEJ-based method, a donor vector harbors gRNA target sites and no HA or microhomology arms (5-25 bp). By contrast, a donor vector in HMEJ-based method harbors gRNA target sites and ∼800 bp HAs. HMEJ is similar to MMEJ, but HMEJ harbors longer and perhaps more stable HAs than MMEJ. This allows targeted integration with a higher efficiency. MMEJ occurs during G1/early S phases, whereas NHEJ occurs throughout the cell cycle. Thus, we speculate that HMEJ may be active during G1/early S phases and single-strand annealing may be involved in this pathway^25^. Elucidation of the precise molecular mechanisms underlying this method requires further studies.

Recently, an NHEJ-based method was developed for efficient gene knock-in *in vivo*^17^. However, our study showed that the NHEJ repair system introduced various types of indel mutations at the junctions, making it difficult to construct endogenous and exogenous fusion genes by in-frame integration for the production of chimeric proteins, consistent with many previous studies^12,13,14,15,18,19,26^. More importantly, NHEJ-based targeted integration can only introduce a donor DNA segment into the cutting site, making it unsuitable for replacing a mutated sequence (such as a point mutation) with the correct one. By contrast, our HMEJ-based strategy introduces a targeted integration in a homology-dependent manner, making DNA segment replacement in the genome practicable. Therefore, this HMEJ-based strategy may offer broader applications in gene therapy. Zhang *et al*.^20^ have reported a similar strategy whereby a double cut of the HR donor by CRISPR/Cas9 could improve knock-in efficiency by 2-5-fold in human iPSCs. In our study, we additionally applied the HMEJ-based method in the construction of transgenic animals including mice and monkeys. Compared with mouse ES cells and N2a cells, we surmise that embryos, cells *in vivo* and human iPSCs may execute both the HR pathway and HMEJ pathway, leading to higher DNA knock-in efficiency using the double-cut donor compared with uncut donor.

Overall, our HMEJ-based method showed robust DNA knock-in in mouse and monkey embryos, markedly reducing the number of animals needed for experiments, especially for non-human primate models^21,23^. With higher editing efficiency and better fidelity compared to the NHEJ-based method^17^, the HMEJ-based method holds a great promise for applications such as *in vivo* targeted gene replacement therapy.

## Materials and Methods

### Animal ethics statement

The use and care of animals complied with the guidelines of the Biomedical Research Ethics Committee at the Shanghai Institutes for Biological Science (CAS), which approved the application entitled “Reproductive physiology of cynomolgus monkey and establishment transgenic monkey” (#ER-SIBS-221106P).

### Construction of plasmids

To generate a single Cas9-sgRNA-EGFP expressing vector, a modified pX330 (Addgene catalog no. 42230) expression vector expressing Cas9-CMV-EGFP and sgRNA was linearized with *Bbs*I digestion, and gel purified. A pair of oligos for each targeting site were phosphorylated, annealed and ligated to the linearized pX330.

To construct the HMEJ donor for *Actb* gene (Supplementary information, Data S1), donor DNA (800 bp HAL-p2A-mCherry-800 bp HAR) sandwiched by 23 nt *Actb*-sgRNA target sequence, U6-*Actb*-sgRNA expression cassette and EF1a-EGFP expression cassettes were subcloned between ITRs of pAAV vector (Addgene catalog no. 37083).

To construct the HR donor for the mouse *Actb* gene (Supplementary information, Data S1), mCherry, EF1a-EGFP, 5′ and 3′ HAs (800 bp) were amplified from pAAV-Ef1a-DIO-mCherry-WPRE-pA (Addgene catalog no. 37083), CAG-GFP-IRES-CRE (Addgene catalog no. 48201) or mouse genome, then subcloned donor (800 bp HAL-p2A-mCherry-800 bp HAR), U6-*Actb*-sgRNA expression cassette and EF1a-EGFP expression cassettes between ITRs of the pAAV vector (Addgene catalog no. 37083).

To construct the MMEJ donor for the *Actb* gene (Supplementary information, Data S1), donor DNA (HAL-p2A-mCherry-HAR) sandwiched by 23 nt *Actb*-sgRNA target sequence, U6-*Actb*-sgRNA expression cassette and EF1a-EGFP expression cassettes were subcloned between ITRs of the pAAV vector (Addgene catalog no. 37083).

To construct the NHEJ donor for the *Actb* gene (Supplementary information, Data S1)^17^, donor DNA (p2A-mCherry) sandwiched by 23 nt *Actb*-sgRNA target sequence, U6-*Actb*-sgRNA expression cassette and EF1a-EGFP expression cassettes were subcloned between ITRs of the pAAV vector (Addgene catalog no. 37083).

The resulting fragment, or linearized vector was purified with a Gel Extraction Kit (Omega, D2500-02) and concentrated by ethanol precipitation. All the plasmid constructs were extracted using the Plasmid Midi Kit (Qiagen, 12143) and verified by DNA sequencing.

### Cell culture and transfection

Mouse ESCs (129/Sv × C57BL/6 ES cell and E14 cell) were cultured in 2i medium, comprising Dulbecco's modified Eagle's Medium (DMEM) (Gibco, 11965-092) containing 15% fetal bovine serum (FBS) (Gibco), 1 000 U/ml mouse Lif, 2 mM glutamine (Sigma), 1% penicillin/streptomycin (Thermo Fisher Scientific), 0.1 mM β-mercaptoethanol (Sigma), 0.1 mM non-essential amino acids (Gibco),1 μM PD0325901 and 3 μM CHIR99021. Monkey COS-7 cells were cultured in DMEM (Gibco) containing 10% FBS (Gibco). All cells were cultured at 37 °C in a 5% CO_2_ atmosphere. Mouse ESCs were transfected using Lipofectamine 3000 Reagent (Invitrogen) according to the manufacturer's instructions. For each well of a six-well plate, a total of 5 μg plasmids (Cas9: donor = 1:1) was used. After 48 h, transfection-positive ES cells were sorted into six-well plates using BD FACS Aria II for further culture and analysis.

Primary cultures of astrocytes were prepared as described previously^27^. Primary astrocytes were obtained from the dorsal midbrain of P5-P7 mice and were cultured in a medium consisting of DMEM/F-12, 10% FBS (Invitrogen), penicillin/streptomycin (Invitrogen) and supplemented with B27 (Invitrogen), 10 ng/ml epidermal growth factor (EGF), and 10 ng/ml fibroblast growth factor 2 (FGF2). Primary neurons were obtained from the cortex of E14.5 C57 mouse brains and plated at a density of 2 × 10^5^ cells per well onto glass coverslips coated with poly-𝒟-Lysine and pre-incubated in medium containing 5% FBS. After 1 h, culture medium was changed to serum-free Neurobasal medium with 2% B27 (Invitrogen), 1% Glutamax (Invitrogen) and 1% penicillin/streptomycin (Thermo Fisher Scientific). All cells were cultured at 37 °C with 5% CO_2_ incubation. One half of the volume of culture media was replaced every 3 days.

Lentivirus was packaged by transfecting HEK293T cells using polyethylenimine at a final concentration of 50 μg/ml, the ratio of donor/sgRNA (HR/NHEJ/MMEJ/HMEJ) and packaging vectors psPAX2 (Addgene 12260) and pMD2.G (Addgene 12259) is 4:3:2, respectively. Virus supernatant was collected 2-3 days post transfection. Astrocytes and neurons were infected with a mixture of lenti-donor/sgRNA and lenti-spCas9 virus (Supplementary information, Figure S4A and S4B).

For comparison of different knock-in strategies, treatment with 10 μM Nu7026 (Selleck), 1 μM Scr7 (Selleck) or 4 mM caffeine (Sigma Aldrich) was started 1 day before transfection and was continued until 2 days after transfection.

### Production of Cas9 mRNA and sgRNA

T7 promoter was added to the Cas9 coding region by PCR amplification of px260, using primer Cas9 F and R (Supplementary information, Table S2). T7-Cas9 PCR product was purified and used as the template for *in vitro* transcription (IVT) using mMESSAGE mMACHINE T7 ULTRA kit (Life Technologies). T7 promoter was added to the sgRNA template by PCR amplification of px330, using the primers listed in Supplementary information, Table S2. The T7-sgRNA PCR product was purified and used as the template for IVT using the MEGA shortscript T7 kit (Life Technologies). Both the Cas9 mRNA and the sgRNAs were purified using the MEGA clear kit (Life Technologies) and eluted in RNase-free water.

### Zygote injection, embryo culturing and embryo transplantation

For the gene editing of mice, super ovulated female B6D2F1 (C57BL/6 × DBA2J) mice (7-8 weeks old) were mated to B6D2F1 males, and fertilized embryos were collected from the oviducts. Cas9 mRNA (100 ng/μl), sgRNA (50 ng/μl) and donor vector (100 ng/l) were mixed and injected into the cytoplasm of fertilized eggs with well-defined pronuclei in a droplet of HEPES-CZB medium containing 5 μg/ml cytochalasin B using a FemtoJet microinjector (Eppendorf) with constant flow settings. The injected zygotes were cultured in KSOM medium with amino acids at 37 °C under 5% CO_2_ in air to blastocysts for fluorescence observation. For generation of knock-in mice, the injected zygotes were cultured to the two-cell stage and 25-30 two-cell embryos were transferred into the oviducts of pseudopregnant ICR females at 0.5 dpc.

For the gene editing of *Macaca fascicularis* monkeys, laparoscopy was used for oocyte collection. Oocytes were aspirated from follicles 2-8 mm in diameter, about 32-36 h after hCG stimulation^28^. The collected oocytes were cultured in pre-equilibrated maturation medium. Metaphase II arrested oocytes were used to perform intracytoplasmic sperm injection, and fertilization was confirmed by the presence of two pronuclei. The zygotes were injected with Cas9 mRNA (100 ng/μl), sgRNAs (50 ng/μl) and HMEJ donor (50 or 100 ng/μl). After injection, the embryos were cultured in HECM-9 medium for 7 days to the morula/blastocyst stage and harvested for genome extraction and analysis.

### *In utero* electroporation

The experimental procedures for *in utero* electroporation have been described previously^29^. E14.5 pregnant C57BL/6 mice were anaesthetized with pentobarbital sodium (50 mg/kg, Sigma). The final concentration of each plasmid (EFs-spCas9-NLS-SV40polyA, the donor vector for HR, NHEJ, MMEJ or HMEJ) was 2 μg/μl. Plasmids were injected into the embryos' lateral ventricles with 0.005% fast green solution (Sigma). For electroporation, five pulses of 50-ms duration separated by 950-ms intervals were applied at 35 V using ECM 830 (BTX). The uterine horns then were placed back into the abdominal cavity and allowed to develop *in utero* for the indicated time.

### Hydrodynamic injection and hepatocyte isolation

Vectors for hydrodynamic tail vein injection were prepared using the EndoFree-Midi Kit (Qiagen). For hydrodynamic liver injection, plasmid DNA suspended in 2 ml saline was hyperdynamically injected into 8-week-old male/female mice (C57BL/6J) via the tail vein in 5-7 s. The amount of injected DNA was 30 μg each for HDR/NHEJ/MMEJ/ HMEJ donor + spCas9. An equal amount of HDR/NHEJ/MMEJ/HMEJ donor only was used as a control for each experiment. C57 mice were killed at 5-9 days post injection. Separated liver lobes were harvested for either genomic DNA extraction or fixed with 4% of paraformaldehyde. For hepatocyte isolation, primary mouse hepatocytes were isolated using a standard two-step collagenase perfusion method^30^. Hepatocytes were purified by low-speed centrifugation (1 000 rpm., 10 min) through 40% Percoll (Sigma).

### Stereotaxic AAV injection in adult brain

Eight-week-old C57BL/6 mice received AAV9 injections. We injected a 1:1 mixture of AAV9-spCas9 and AAV9-HMEJ for HMEJ-mediated-targeted *Actb*-2A-mCherry knock-in. As a control, a 1:1 mixture of AAV9-HMEJ and PBS buffer was used (Figure 5G and 5H). AAV9 was injected into the cerebral cortex (V1) using the following coordinates: 3.4 mm rostral, 2.6 mm lateral relative to the bregma and 0.5-0.8 mm ventral from the pia. The injected brain regions were dissected for immunostaining or DNA extraction 3 weeks post injection.

### Immunostaining

Mice were transcardially perfused with 0.9% saline followed by 4% paraformaldehyde using a peristaltic pump (Gilson) and fixed overnight at 4 °C. The tissue was then dehydrated using 30% sucrose until it sank to the bottom of tube. Tissue sections were taken on a Leica CM1950-Cryostat (Leica) at a thickness of 40 μm for brain, 10 μm for liver. Sections were rinsed three times in 0.1M phosphate buffer (PB) and incubated with the primary antibodies: rabbit anti-mCherry (1:3 000, GeneTex), chicken anti-GFP (1:1000,Invitrogen),mouse anti-NeuN (1:3000,Sigma), which were diluted in diluent with 5% NGS overnight at 4 °C. The following day, sections were washed three times in PB and then incubated with the secondary antibodies: 561-AffiniPure Goat Anti-Rabbit IgG (1:500,Jackson Immunoresearch), 488-AffiniPure Goat Anti-Chicken IgG (1:500, Invitrogen) and Cy5-AffiniPure Goat Anti-Mouse IgG (1:500, Jackson Immunoresearch) for 2 h at room temperature on an orbital shaker. Finally, the sections were counterstained with DAPI for 20 min and mounted with SlowFade Diamond Antifade Mountant (Life) on glass slides.

### Embryo genotyping analysis

For picking up and transferring single embryos, we used a glass capillary under a dissection microscope. Single embryos were picked up based on fluorescence, and transferred directly into PCR tubes containing 1.5 μl lysis buffer (0.1% tween 20, 0.1% Triton X-100 and 4 μg/ml proteinase K). The samples were incubated for 30 min at 56 ˚C and heat inactivate proteinase K at 95 ˚C for 10 min. PCR ampliﬁcation was performed using nested primer sets (Supplementary information, Table S2). ExTaq was activated at 95 °C for 3 min, and PCR was performed for 30 cycles at 95 °C for 30 s, 60 °C for 30 s and 72 °C for 1 min, with a final extension at 72 °C for 5 min. Secondary PCR was performed using 1 μl primary PCR product and a nested inner primer. PCR was carried out in the same reaction mixture. The PCR product was gel purified and sequenced.

### Statistical analysis

All statistical values are presented as mean ± SEM. Differences between data sets were judged to be significant at *P* < 0.05.

## Author Contributions

XY designed and performed experiments. XW designed and performed plasmid construction and genotyping. XH performed *in utero* electroporation. ZL, YW, C-CZ and Y-HN performed monkey experiments. JL performed hydrodynamic injection. HZ performed stereotaxic AAV injection. YW, WQY, XS, QW, WY and ZH performed mouse experiments. SL and LC analyzed *Dbh* mice. PH designed and supervised experiments in the liver. QS designed and supervised experiments in monkey. LS designed and supervised experiments in mouse. HY supervised the project, designed experiments and wrote the paper.

## Competing Financial Interests

The authors declare no competing financial interests.

## Supplementary Material

Supplementary information, Figure S1 Genotyping analysis of cells with HMEJ- and HR-mediated targeted integration at *Actb* and *Tubb3* loci in mouse ES cells.

Supplementary information, Figure S2 Schematic overview of targeting loci and experimental design in mouse ES cells.

Supplementary information, Figure S3 Knock-in efficiencies of HMEJ donors with different length of HA in mouse ES cells and N2a cells.

Supplementary information, Figure S4 Schematic overview of targeted *Actb*-2A-mCherry knock-in via lentivirus delivery in primary astrocytes and neurons.

Supplementary information, Figure S5 Sequence analysis of blastocysts from *Actb* knock-in by different strategies.

Supplementary information, Figure S6 Sequence analysis of blastocysts from HMEJ-mediated targeted integration at *Nanog*, *Sox2*, and *Cdx2* loci in mouse embryos.

Supplementary information, Figure S7 Gene-edited mice generated by HMEJ-mediated targeted integration at *Dbh* and *Sox2* loci.

Supplementary information, Figure S8 HMEJ-mediated targeted integration at *Actb* loci in COS-7 cells.

Supplementary information, Figure S9 Sequence analysis of hepatocytes after 2A-mcherry knock-in into *Actb* locus by HMEJ-mediated method.

Supplementary information, Table S1 Knock-in mice generated by HMEJ-mediated targeted integration

Supplementary information, Table S2 Primers used in this study.

Supplementary information, Data S1 Plasmids sequences used in this study.
