# Supplementary material for: Homology-mediated end joining-based targeted integration using CRISPR/Cas9
Source: Cell Res. 2017 May 19;27(6):801–14. doi: 10.1038/cr.2017.76 (PMC5518881; doi:10.1038/cr.2017.76)
Supplement: Supplementary information, Figure S1 — Genotyping analysis of cells with HMEJ- and HR-mediated targeted integration at Actb and Tubb3 loci in mouse ES cells. [file cr201776x1.pdf]

Supplementary Figure 1.

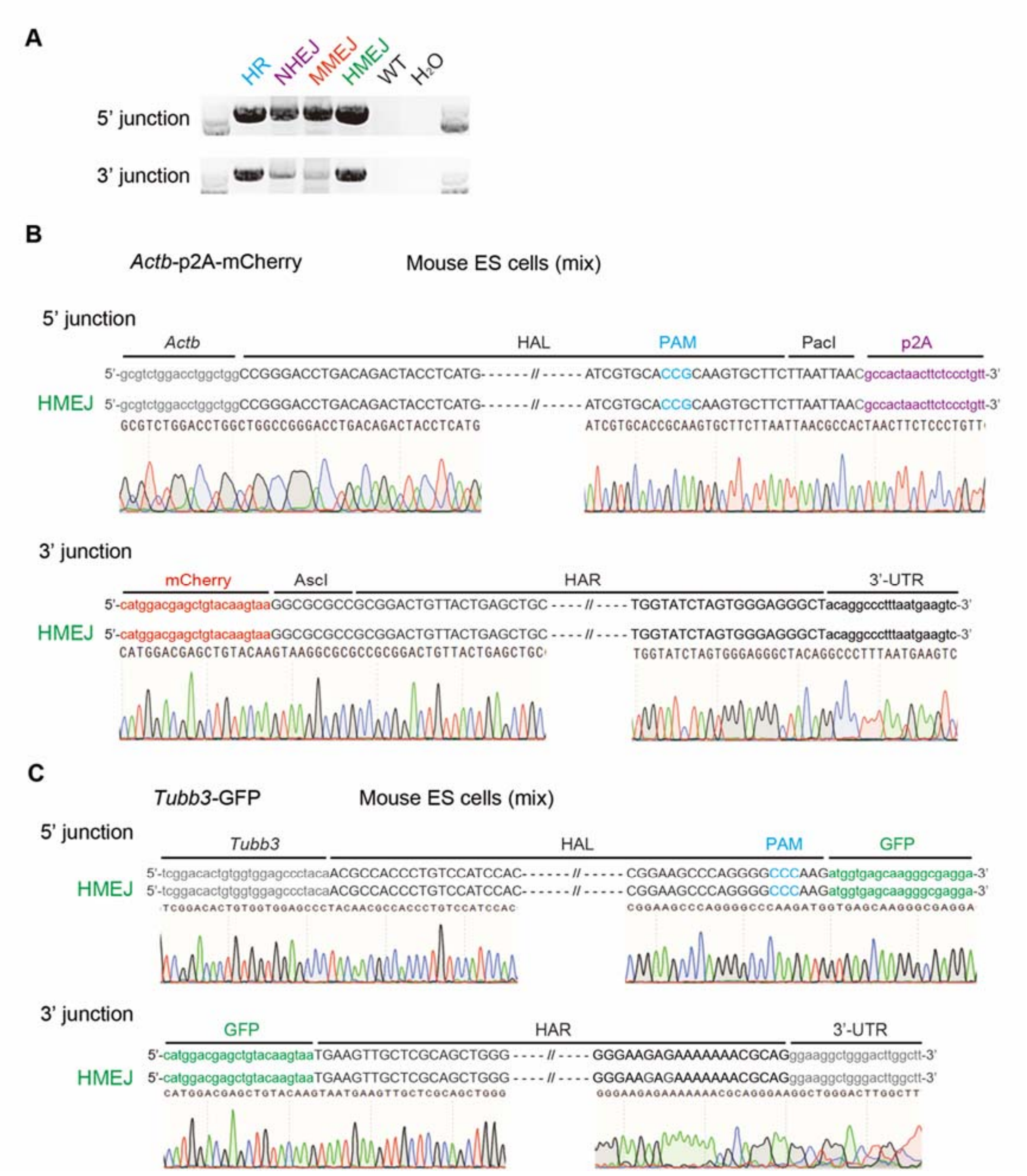

Supplementary Figure 1. Genotyping analysis of cells with HMEJ- and HR-mediated targeted integration at *Actb* and *Tubb3* loci in mouse ES cells**.** PCR products amplified from 5' and 3' junction sites were sequenced. Upper: homology arm; purple, p2A; red, mCherry; green, GFP; blue, PAM sequence.
