# Supplementary material for: Homology-mediated end joining-based targeted integration using CRISPR/Cas9
Source: Cell Res. 2017 May 19;27(6):801–14. doi: 10.1038/cr.2017.76 (PMC5518881; doi:10.1038/cr.2017.76)
Supplement: Supplementary information, Figure S2 — Schematic overview of targeting loci and experimental design in mouse ES cells. [file cr201776x2.pdf]

Supplementary Figure 2.

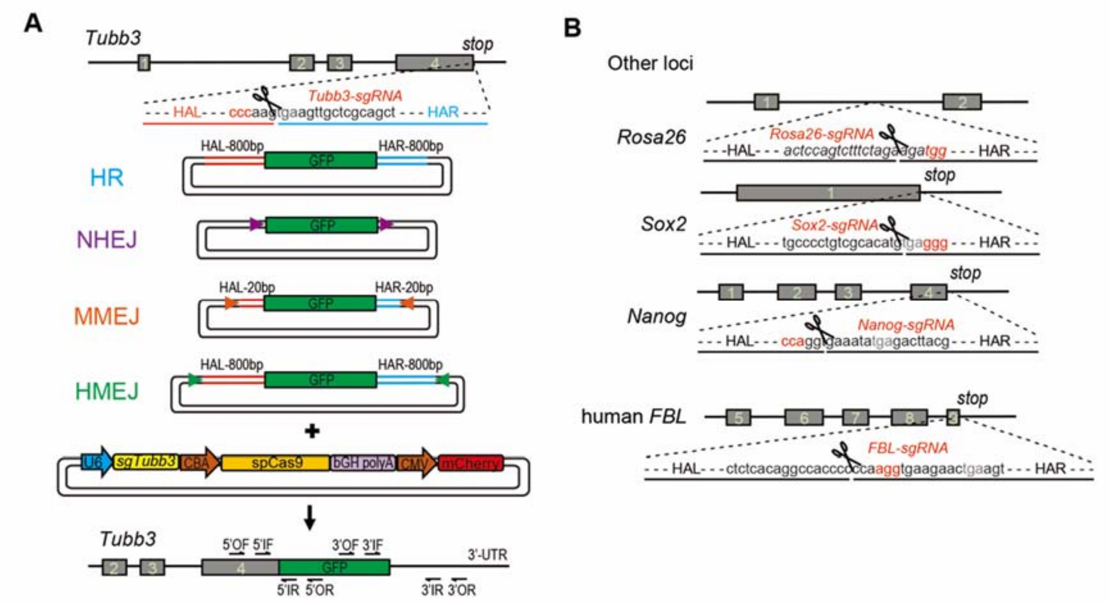

Supplementary Figure 2. Schematic overview of targeting loci and experimental design in mouse ES cells. (**A**) Schematic overview of four gene targeting strategies at *Tubb3* locus. GFP was designed to insert at the last codon of *Tubb3* gene to fuse with *Tubb3*. (**B**) Schematic overview of targeting loci, including mouse genes *Rosa26*, *Nanog*, *Sox2* and human gene *FBL*.
