# Supplementary material for: Homology-mediated end joining-based targeted integration using CRISPR/Cas9
Source: Cell Res. 2017 May 19;27(6):801–14. doi: 10.1038/cr.2017.76 (PMC5518881; doi:10.1038/cr.2017.76)
Supplement: Supplementary information, Figure S3 — Knock-in efficiencies of HMEJ donors with different length of HA in mouse ES cells and N2a cells. [file cr201776x3.pdf]

Supplementary Figure 3.

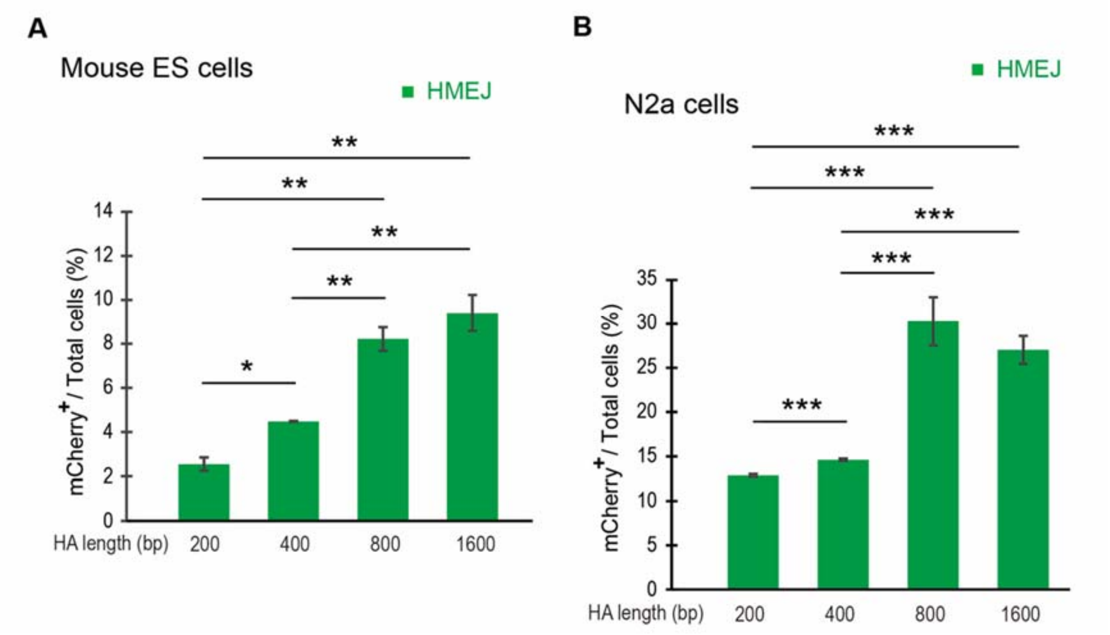

Supplementary Figure 3. Knock-in efficiencies of HMEJ donors with different length of HA in mouse ES cells and N2a cells. (**A**-**B**) A series of HMEJ donors with HA in the length of 200, 400, 800 or 1,600 bp for p2A-mCherry knock-in at *Actb* locus, together with Cas9/sgRNA, were transfected into mouse ES cells (**A**) or N2a cells (**B**), and knock-in efficiencies were measured by FACS. Results were presented as mean ± s.d. *P < 0.05, **P < 0.01, ***P < 0.001, unpaired Student's *t*-test
