# Supplementary material for: Homology-mediated end joining-based targeted integration using CRISPR/Cas9
Source: Cell Res. 2017 May 19;27(6):801–14. doi: 10.1038/cr.2017.76 (PMC5518881; doi:10.1038/cr.2017.76)
Supplement: Supplementary information, Figure S4 — Schematic overview of targeted Actb-2A-mCherry knock-in via lentivirus delivery in primary astrocytes and neurons. [file cr201776x4.pdf]

Supplementary Figure 4.

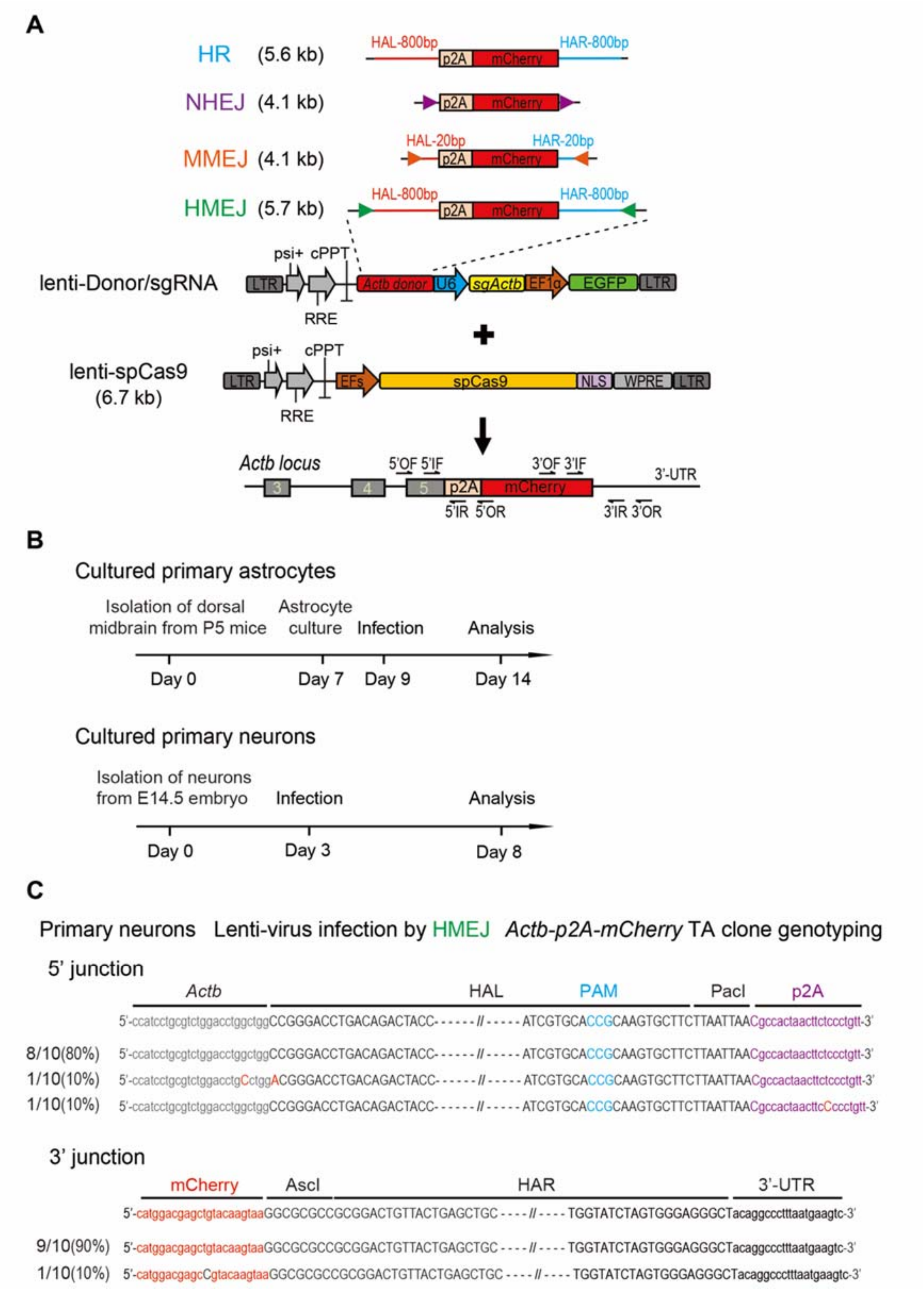

Supplementary Figure 4. Schematic overview of targeted *Actb*-2A-mCherry knock-in via lentivirus delivery in primary astrocytes and neurons. (**A**) Schematic overview of four types of donor/sgRNAs and Cas9 using lentivirus for p2A-mCherry knock-in at the last codon of *Actb* gene. HAL/HAR, left/right homology arm; triangles, sgRNA target sites;

OF/OR, outer forward/reverse primer; IF/IR, inner forward/reverse primer. (**B**) Experimental scheme for targeted *Actb*-2A-mCherry knock-in in primary astrocytes and neurons. (**C**) Genotyping analysis of cells with HMEJ-mediated targeted integration at *Actb* locus in primary neurons**.** PCR products amplified from 5’ and 3’ junction sites were TA cloned and sequenced. Upper, homology arm; purple, p2A; red, mCherry; blue, PAM sequence.
