# Supplementary material for: Homology-mediated end joining-based targeted integration using CRISPR/Cas9
Source: Cell Res. 2017 May 19;27(6):801–14. doi: 10.1038/cr.2017.76 (PMC5518881; doi:10.1038/cr.2017.76)
Supplement: Supplementary information, Figure S5 — Sequence analysis of blastocysts from Actb knock-in by different strategies. [file cr201776x5.pdf]

**Supplementary Figure 5.**

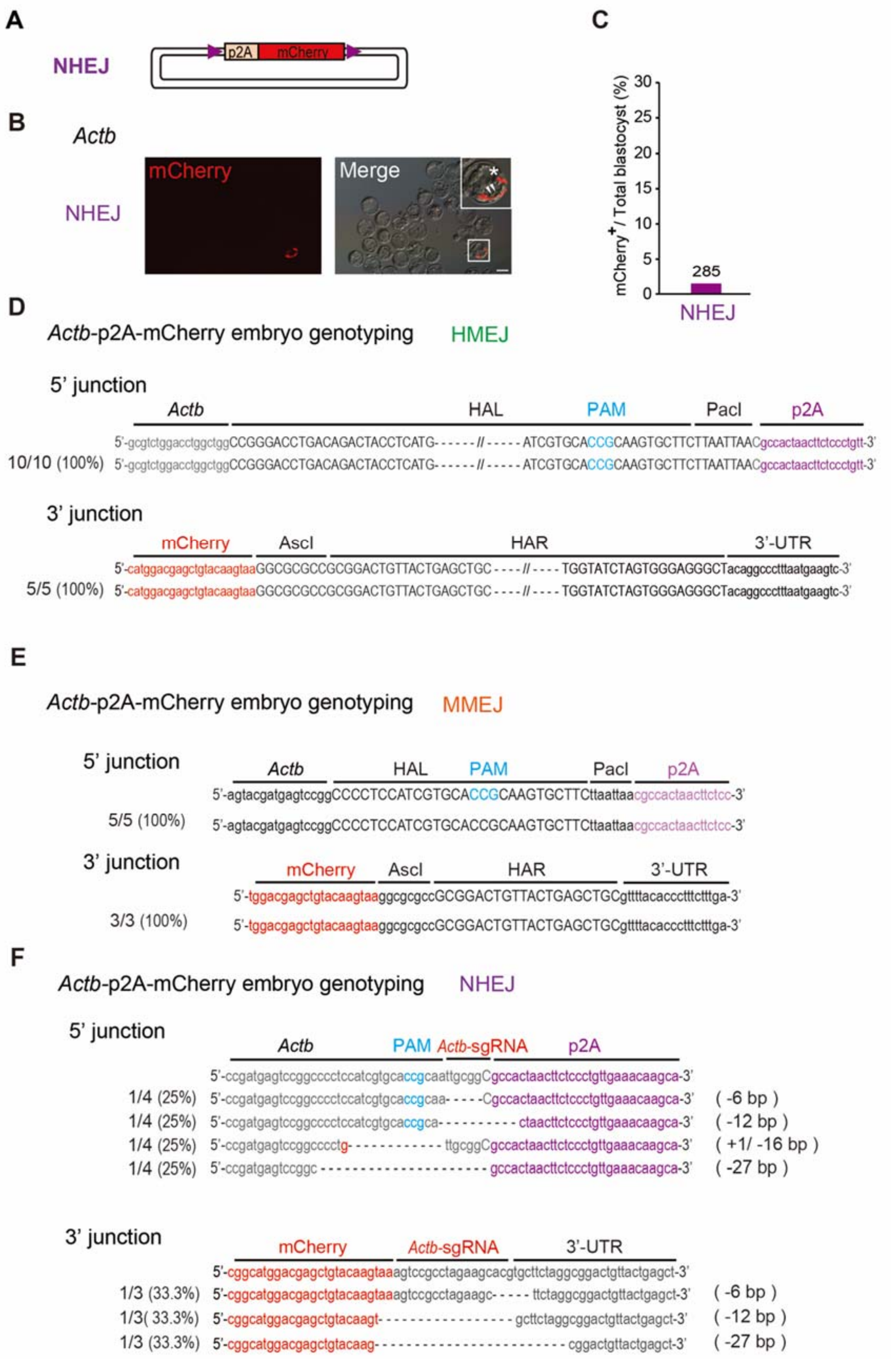


**Supplementary Figure 5.** Sequence analysis of blastocysts from *Actb* knock-in by different strategies. (**A**) Schematic overview of NHEJ donor vector. (**B**) Representative

immunofluorescence images of gene-edited blastocysts by NHEJ-mediated method. Inset, higher magnification image. (**C**) Knock-in efficiency by NHEJ-mediated method indicated by percentage of $mCherry^+$ blastocysts. Number above each bar, total blastocysts counted. (**D-F**) Sequence analysis of $mCherry^+$ blastocysts from HMEJ- (**D**), MMEJ- (**E**) and NHEJ- (**F**) mediated targeting. PCR products amplified from individual $mCherry^+$ blastocyst at 5' and 3' junction sites were sequenced. Upper, homology arm; purple, p2A; red, mCherry; blue, PAM sequence; HAR or HAL, right or left homologous arm. Dashed lines mark the region omitted for clarity.
