# Supplementary material for: Homology-mediated end joining-based targeted integration using CRISPR/Cas9
Source: Cell Res. 2017 May 19;27(6):801–14. doi: 10.1038/cr.2017.76 (PMC5518881; doi:10.1038/cr.2017.76)
Supplement: Supplementary information, Figure S6 — Sequence analysis of blastocysts from HMEJ-mediated targeted integration at Nanog, Sox2, and Cdx2 loci in mouse embryos. [file cr201776x6.pdf]

Supplementary Figure 6.

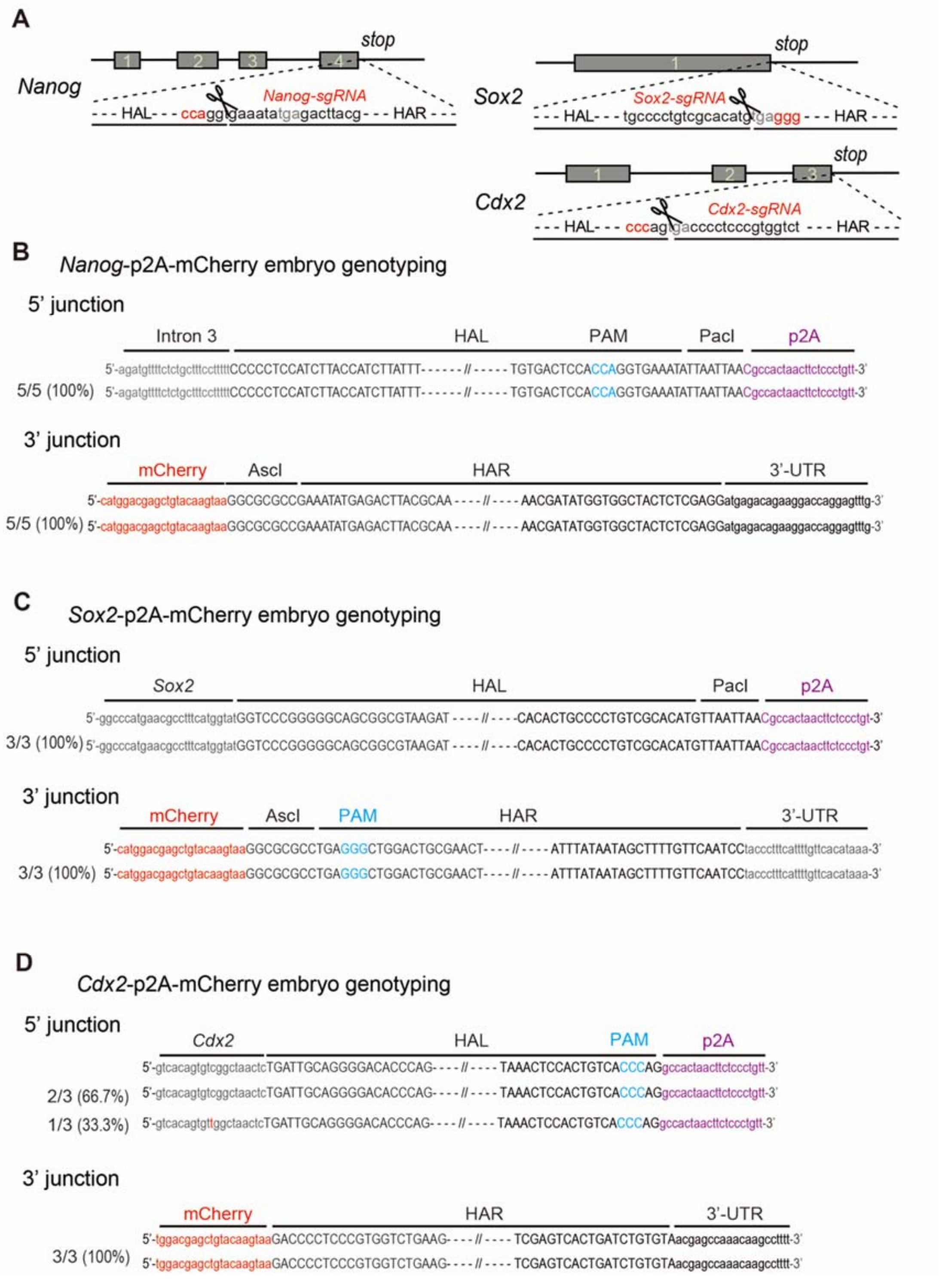

Supplementary Figure 6. Sequence analysis of blastocysts from HMEJ-mediated targeted integration at *Nanog, Sox2*, and *Cdx2* loci in mouse embryos. (**A**) Schematic overview of HMEJ-mediated gene targeting strategy at *Sox2*, *Nanog* and *Cdx2* loci in mouse embryos. (**B**-**D**) Sequence analysis of mCherry^{+} blastocysts from HMEJ-mediated targeting at *Nanog, Sox2*, and *Cdx2* loci. PCR products amplified from individual blastocyst on 5' and 3' junction sites were sequenced. Upper, homology arm; purple,

p2A; red, mCherry; blue, PAM sequence; HAR or HAL, right or left homologous arm. Dashed lines mark the region omitted for clarity.
