# Supplementary material for: Homology-mediated end joining-based targeted integration using CRISPR/Cas9
Source: Cell Res. 2017 May 19;27(6):801–14. doi: 10.1038/cr.2017.76 (PMC5518881; doi:10.1038/cr.2017.76)
Supplement: Supplementary information, Figure S7 — Gene-edited mice generated by HMEJ-mediated targeted integration at Dbh and Sox2 loci. [file cr201776x7.pdf]

Supplementary Figure 7.

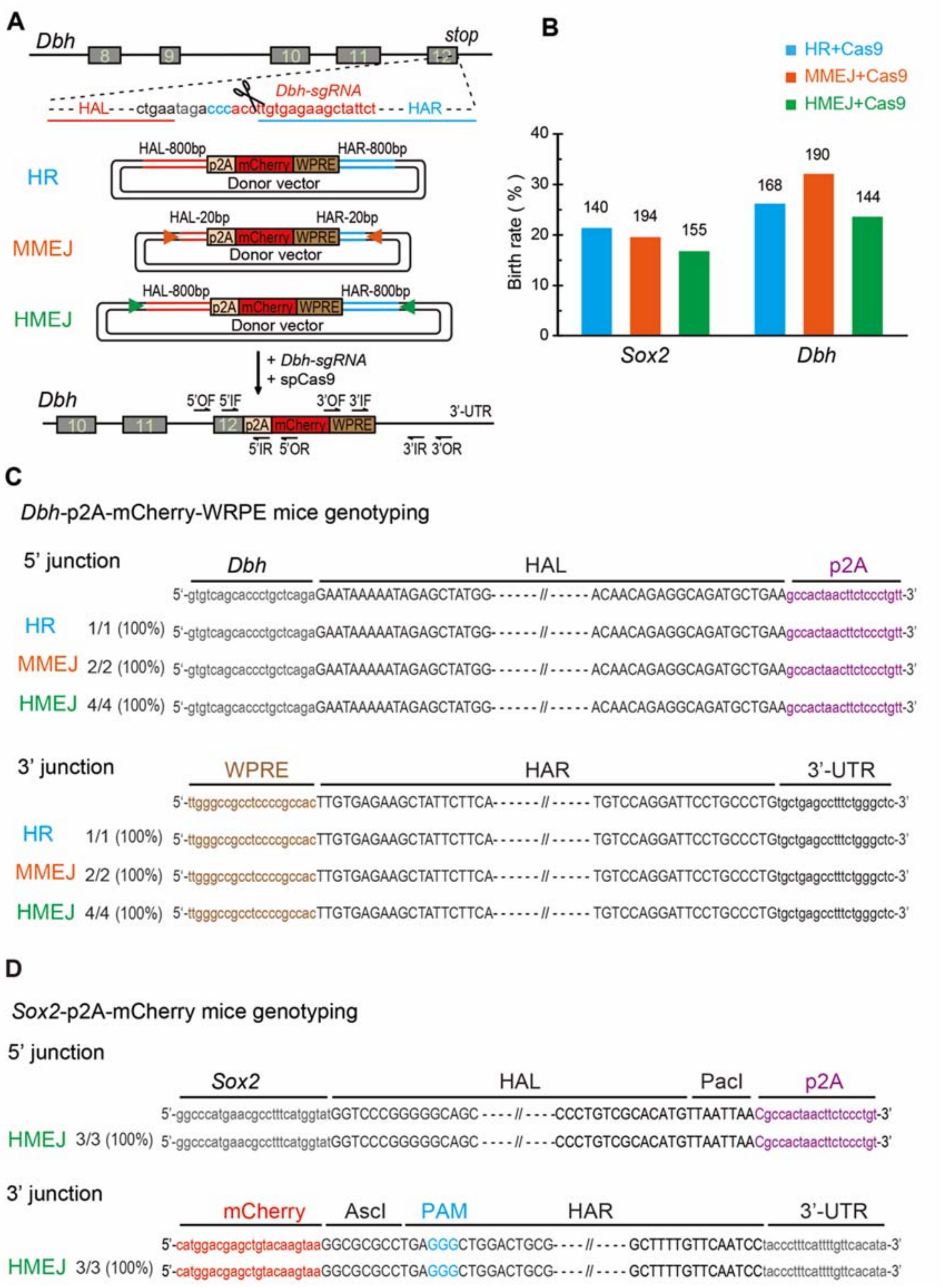

Supplementary Figure 7. Gene-edited mice generated by HMEJ-mediated targeted integration at *Dbh* and *Sox2* loci. (**A**) Schematic overview of gene-edited mice generation with HR-, MMEJ-, and HMEJ-based strategy at *Dbh* locus. (**B**) Birth rate of gene-edited mice by HR-, MMEJ- and HMEJ-based method. Number above each bar, total embryos transferred. (**C**-**D**) Sequence analysis of gene-edited mice at *Dbh* and *Sox2* locus. PCR products amplified from 5' and 3' junction sites were sequenced. Upper, homology arm;

purple, p2A; red, mCherry; blue, PAM sequence; HAR or HAL, right or left homologous arm. Dashed lines mark the region omitted for clarity.
