# Supplementary material for: Homology-mediated end joining-based targeted integration using CRISPR/Cas9
Source: Cell Res. 2017 May 19;27(6):801–14. doi: 10.1038/cr.2017.76 (PMC5518881; doi:10.1038/cr.2017.76)
Supplement: Supplementary information, Figure S8 — HMEJ-mediated targeted integration at Actb loci in COS-7 cells. [file cr201776x8.pdf]

Supplementary Figure 8.

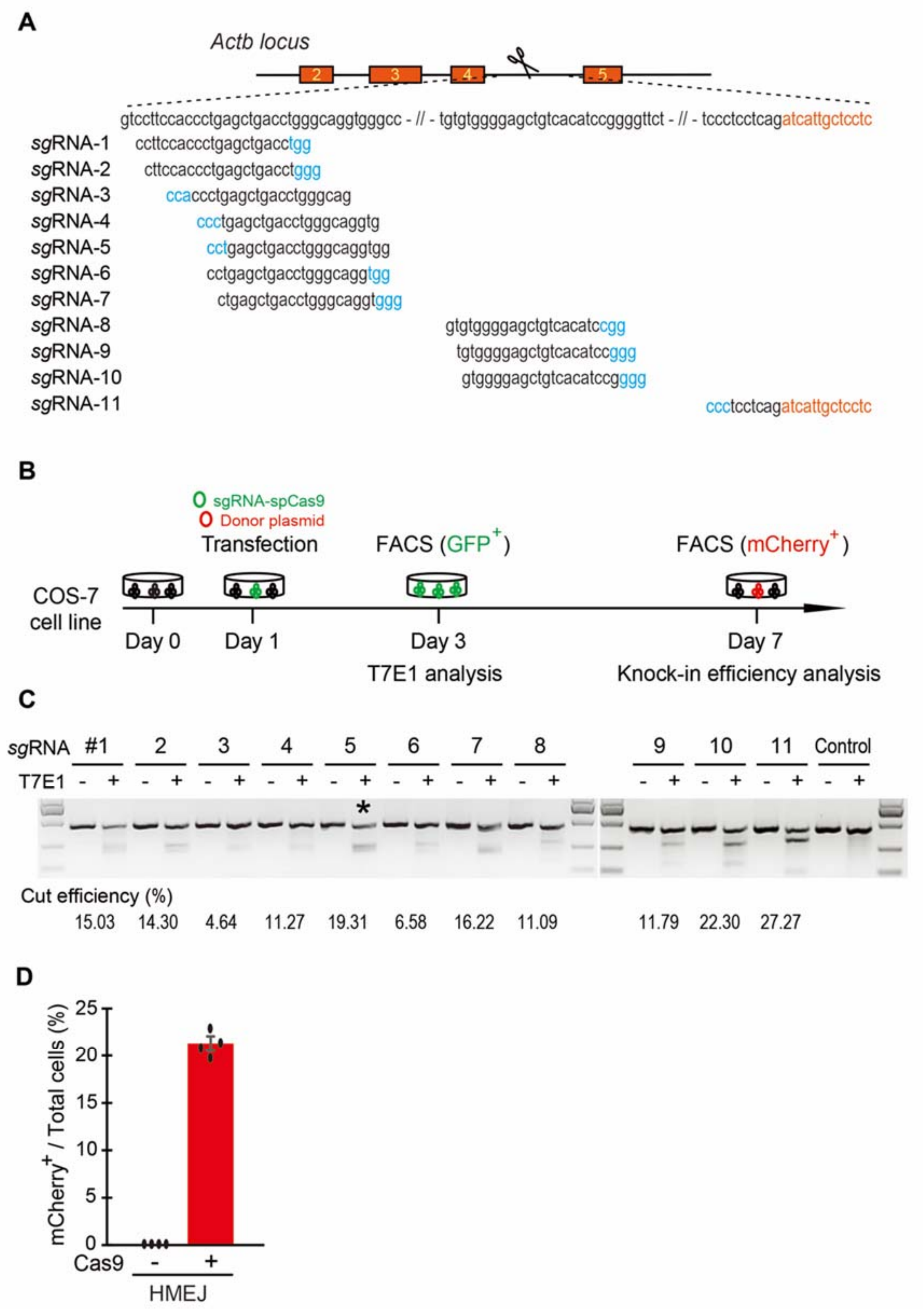

Supplementary Figure 8. HMEJ-mediated targeted integration at *Actb* loci in COS-7 cells. (**A**-**B**) Experimental scheme for targeted *Actb*-2A-mCherry knock-in in COS-7 cells. 11 different sgRNA plasmids, with Cas9 and GFP expression, were individually transfected into COS-7 cells and GFP^{+} cells were sorted at day 3 for T7E1 assay. The sgRNA with relatively higher cleavage efficiency was also transfected with donor vector

for *Actb*-2A-mCherry targeted integration. (**C**) T7E1 assay for *Actb* targeting. The control is normal COS-7 cell genomic DNA. *, the sgRNA used for *Actb*-2A-mcherry knock-in experiment. (**D**) Relative knock-in efficiency of *Actb*-p2A-mCherry knock-in in COS-7 cells measured by the percentage of mCherry^{+} cells among transfected cells. Results were presented as mean ± s.d. The input data points were shown as black dots.
