# Supplementary material for: Homology-mediated end joining-based targeted integration using CRISPR/Cas9
Source: Cell Res. 2017 May 19;27(6):801–14. doi: 10.1038/cr.2017.76 (PMC5518881; doi:10.1038/cr.2017.76)
Supplement: Supplementary information, Figure S9 — Sequence analysis of hepatocytes after 2A-mcherry knock-in into Actb locus by HMEJ-mediated method. [file cr201776x9.pdf]

**Supplementary Figure 9.**

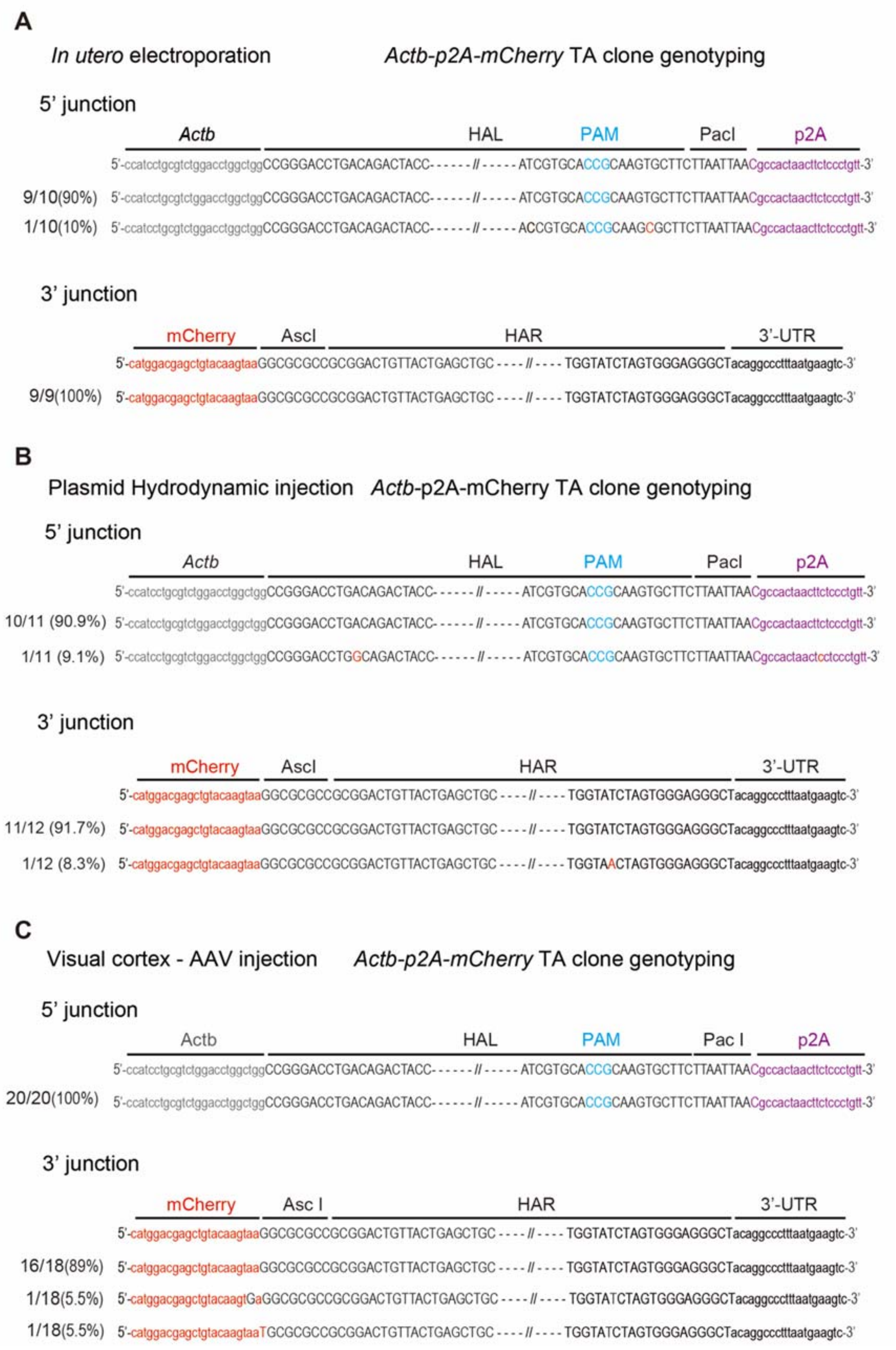


**Supplementary Figure 9.** Sequence analysis of hepatocytes after 2A-mcherry knock-in into *Actb* locus by HMEJ-mediated method. (**A**-**C**) Genotyping analysis of cells with

HMEJ-mediated knock-in via *in utero* electroporation (**A**), hydrodynamic injection (**B**) and AAV injection (**C**). PCR products amplified from 5’ and 3’ junction sites were TA cloned and sequenced. The HMEJ intended knock-in sequence is shown at the top. Upper, homology arm; purple, p2A; red, mCherry. The PAM sequence is highlighted in blue.
