# Supplementary material for: Homology-mediated end joining-based targeted integration using CRISPR/Cas9
Source: Cell Res. 2017 May 19;27(6):801–14. doi: 10.1038/cr.2017.76 (PMC5518881; doi:10.1038/cr.2017.76)
Supplement: Supplementary information, Table S1 — Knock-in mice generated by HMEJ-mediated targeted integration [file cr201776x10.pdf]

Supplementary information, Table S1. Knock-in mice generated by HMEJ-mediated targeted integration

Gene	Method	Transferred Embryos (recipients)	Newborns (birth rate%)	5’&3’ junction (%)	Precise integration (%)	Germline transmission (%)
Dbh	HR	168 (6)	44 (26.2)	1/44 (2.27)	1/1 (100)	1/1 (100)
	MMEJ	190 (7)	61 (32.1)	2/61 (3.28)	2/2 (100)	2/2 (100)
	HMEJ	144 (6)	34 (23.6)	4/33 (12.1)	4/4 (100)	4/4 (100)
Sox2	HR	140 (6)	30 (21.4)	1/30 (3.33)	1/1 (100)	N.A.
	MMEJ	194 (7)	38 (19.6)	3/38 (7.9)	3/3 (100)	N.A.
	HMEJ	155 (6)	26 (16.8)	7/26 (26.9)	7/7 (100)	N.A.

Cas9 mRNA (100 ng/μl), sgRNAs (50 ng/μl), and donor vectors (100 ng/μl) were injected into fertilized eggs. 2-cell embryos derived from the injected embryos were transferred into recipients and newborn pups were obtained and genotyped.
