# Supplementary material for: Homology-mediated end joining-based targeted integration using CRISPR/Cas9
Source: Cell Res. 2017 May 19;27(6):801–14. doi: 10.1038/cr.2017.76 (PMC5518881; doi:10.1038/cr.2017.76)
Supplement: Supplementary information, Table S2 — Primers used in this study. [file cr201776x11.pdf]

Supplementary information, Table S2. Primers used in this study.

**Cas9 mRNA and sgRNA primer for in vitro transcription (IVT)**	
Actb T7_F	TAATACGACTCACTATAGGGagtccgcctagaagcacttg
Nanog T7_F	TAATACGACTCACTATAGGGcgtaagtctcatatttcacc
Sox2 T7_F	TAATACGACTCACTATAGGGtgcccctgtcgcacatgtga
Cdx2 T7_F	TAATACGACTCACTATAGGGagaccacgggaggggtcact
Dbh T7_F	TAATACGACTCACTATAGGGagaatagcttctcacaaggt
Macaca-*Actb* T7_F	TAATACGACTCACTATAGGGccacctgcccaggtcagctc
sgRNA T7_R	AAAAGCACCGACTCGGTGCC
Cas9 IVT_F	TAATACGACTCACTATAGGGagatttcaggttggaccggtg
Cas9 IVT_R	GACGTCAGCGTTCGAATTGC

***Actb* identification primer**	
5'Outer_F	CCCTGAGTGTTTCTTGTGGC
5'Outer_R	TGGAGCCGTACATGAACTGA
5'Inner_F	CCATCTACGAGGGCTATGCT
5'Inner_R	TGAAGCGCATGAACTCCTTG
3'Outer_F	GCCCCGTAATGCAGAAGAAG
3'Outer_R	AGGTAGTGTTAGTGCAGGCC
3'Inner_F	CTACGACGCTGAGGTCAAGA
3'Inner_R	GCCTAGGTTTCTGGAGGAGT
Actb Outer_F	GGCTCCTAGCACCATGAAGA
Actb Outer_R	CCTTCACCGTTCCAGTTTTT
Actb Inner_F	GATCATTGCTCCTCCTGAGC
Actb Inner_R	GAGTCAAAAGCGCCAAAACA

***Nanog* identification primer**	
5'Outer_F	AGGCCTGGCTGTCATGTTTA
5'Outer_R	TGGAGCCGTACATGAACTGA
5'Inner_F	CGAATGTCCTGTCACTCTGC
5'Inner_R	TGAAGCGCATGAACTCCTTG
3'Outer_F	GCCCCGTAATGCAGAAGAAG
3'Outer_R	GACCCTCCCCTTCACATACC
3'Inner_F	TCCCACAACGAGGACTACAC
3'Inner_R	CTGGCATCGGTTCATCATGG

Sox2 identification primer

5'Outer_F	ACCGGCGGCAACCAGAAGAACA
5'Outer_R	TCACCTTCAGCTTGGCGGTC
5'Inner_F	CAACCAGAAGAACAGCCCGGA
5'Inner_R	GGTCTGGGTGCCCTCGTAG
3'Outer_F	GCCCCGTAATGCAGAAGAAG
3'Outer_R	GATTCTCGGCAGCCTGATTC
3'Inner_F	CTACGACGCTGAGGTCAAGA
3'Inner_R	TCGGCAGCCTGATTCCAATA

Cdx2 identification primer

5'Outer_F	ACTTGGACAGAGAAAGAGCGATT
5'Outer_R	TCCATGTGCACCTTGAAGCG
5'Inner_F	AACAAAGGTCCAGTCTACGCAT
5'Inner_R	GGCCATGTTATCCTCCTCGC
3'Outer_F	GACGGCCCCGTAATGCAGAA
3'Outer_R	TAGCTTGCAACCAGAGAAGATGT
3'Inner_F	CTACGACGCTGAGGTCAAGA
3'Inner_R	CGACTTCCCTTCACCATACAAC

Dbh mice identification primer

5'_F	GATGGGGTTAAGTGGTGGGT
5'_R	CACCTTGAAGCGCATGAACT
3'_F	CGTCCCTTCGGCCCTCAATC
3'_R	ACTCCCTTCCAAAACCATCAAAGA

Sox2 mice identification primer

5'_F	CAACCAGAAGAACAGCCCGGA
5'_R	TCACCTTCAGCTTGGCGGTC
3'_F	CTACGACGCTGAGGTCAAGA
3'_R	TCGGCAGCCTGATTCCAATA

Macaca-_Actb_ identification primer

5'Outer_F	CTAACACTGGCTCGTGTGACAA
5'Outer_R	ACCTTGAAGCGCATGAACTCCT
5'Inner_F	GCTGGTGTAAAGCGGCCTTG
5'Inner_R	GATGATGGCCATGTTATCCTCCT

3'Outer_F	GGCGCCTACAACGTCAACATC
3'Outer_R	CTGAATCCTGAATCTTCCCCCA
3'Inner_F	GTTGGACATCACCTCCCACAAC
3'Inner_R	CCCTCTAAGGCTGCTCAATG
Actb_F	CTAAGTCCGCCCTCATTTCTTC
Actb_R	GTCATACTCCTGCTTGCTGATCC

Mecp2 **as reference gene**

Mecp2 Outer_F	ATAACTGGGCCAAACTGTGC
Mecp2 Outer_R	GAGTCGCACATCTGTCTGGA
Mecp2 Inner_F	TGCAGCTTCAGTTCACCTTG
Mecp2 Inner_R	CCACTAACCACAGGCTCCAT

GAPDH **as reference gene**

GAPDH Outer_F	AACTTTGGCATTGTGGAAGG
GAPDH Outer_R	TGTGAGGGAGATGCTCAGTG
GAPDH Inner_F	ACCCAGAAGACTGTGGATGG
GAPDH Inner_R	TGTGAGGGAGATGCTCAGTG
