# Supplementary material for: Homology-mediated end joining-based targeted integration using CRISPR/Cas9
Source: Cell Res. 2017 May 19;27(6):801–14. doi: 10.1038/cr.2017.76 (PMC5518881; doi:10.1038/cr.2017.76)
Supplement: Supplementary information, Data S1 — Plasmids sequences used in this study. [file cr201776x12.pdf]

Supplementary Information, Data S1 Plasmids sequences used in this study.

Plasmid-based donor template sequences (5'-3')

Details of the plasmid-based donor templates are as follows.

Actb HDR donor (upper: homology arm; underline; linker)

CCGGGACCTGACAGACTACCTCATGAAGATCCTGACCGAGCGTGGCTACAGCTTCA
CCACCACAGCTGAGAGGGAAATCGTGCGTGACATCAAAGAGAAGCTGTGCTATGTT
GCTCTAGACTTCGAGCAGGAGATGGCCACTGCCGCATCCTCTTCCTCCCTGGAGAA
GAGCTATGAGCTGCCTGACGGCCAGGTCATCACTATTGGCAACGAGCGGTTCCGAT
GCCCTGAGGCTCTTTTCCAGCCTTCCTTCTTGGGTAAGTTGTAGCCTAGTCCTTTCT
CCATCTAAAGGTGACAAAACTCCTGAGGCCATAGTACAAGTTAAGTCTGATTTCTGTC
ACTCTTCTCTTAGGTATGGAATCCTGTGGCATCCATGAAACTACATTCAATTCCATCAT
GAAGTGTGACGTTGACATCCGTAAAGACCTCTATGCCAACACAGTGCTGTCTGGTGG
TACCACCATGTACCCAGGCATTGCTGACAGGATGCAGAAGGAGATTACTGCTCTGGC
TCCTAGCACCATGAAGATCAAGGTAAGCTAAGCATCCTTAGCTTGGTGAGGGTGGGC
CCTGTGGTTGTCAGAGCAACCTTCTAGGTTTAAGGGGAATCCCAGCACCCAGAGAG
CTCACCATTCACCATCTTGTCTTGCTTTCTTCAGATCATTGCTCCTCCTGAGCGCAAG
TACTCTGTGTGGATCGGTGGCTCCATCCTGGCCTCACTGTCCACCTTCCAGCAGATG
TGGATCAGCAAGCAGGAGTACGATGAGTCCGGCCCCTCCATCGTGCACCGCAAGTG
CTTCttaattaacgccactaacttctccctgttgaaacaagcaggggatgtcgaagagaatcccgggccaatggtgagca
agggcgaggaggataacatggccatcatcaaggagttcatgcgcttcaaggtgcacatggagggctccgtgaacggcca
cgagttcgagatcgagggcgagggcgagggccgcccctacgagggcacccagaccgccaagctgaaggtgaccaag
ggtggcccccctgcccttcgcctgggacatcctgtcccctcagttcatgtacggctccaaggcctacgtgaagcaccccgccg
acatccccgactacttgaagctgtccttccccgagggcttcaagtgggagcgcgtgatgaacttcgaggacggcggcgtgg
tgaccgtgacccaggactcctccctgcaggacggcgagttcatctacaaggtgaagctgcgcggcaccaacttcccctccg
acggccccgtaatgcagaagaagaccatgggctgggaggcctcctccgagcggatgtaccccgaggacggcgccctga
agggcgagatcaagcagaggctgaagctgaaggacggcggccactacgacgctgaggtcaagaccacctacaaggc
caagaagcccgtgcagctgcccggcgcctacaacgtcaacatcaagttggacatcacctcccacaacgaggactacacc
atcgtggaacagtacgaacgcgccgagggccgccactccaccggcggcatggacgagctgtacaagtaaggcgcgcc
GCGGACTGTTACTGAGCTGCGTTTTACACCCTTTCTTTGACAAAACCTAACTTGCGC
AGAAAAAAAAAAAATAAGAGACAACATTGGCATGGCTTTGTTTTTTTAAATTTTTTTTAA
AGTTTTTTTTTTTTTTTTTTTTTTTTTTTAAGTTTTTTTGTTTTGTTTTGGCGCTTTTG
ACTCAGGATTTAAAAACTGGAACGGTGAAGGCGACAGCAGTTGGTTGGAGCAAACA
TCCCCCAAAGTTCTACAAATGTGGCTGAGGACTTTGTACATTGTTTTGTTTTTTTTTTT
TTTTGGTTTTGTCTTTTTTTAATAGTCATTCCAAGTATCCATGAAATAAGTGGTTACAGG
AAGTCCCTCACCCTCCCAAAAGCCACCCCCACTCCTAAGAGGAGGATGGTCGCGTC
CATGCCCTGAGTCCACCCCGGGGAAGGTGACAGCATTGCTTCTGTGTAAATTATGTA
CTGCAAAAATTTTTTTAAATCTTCCGCCTTAATACTTCATTTTTGTTTTTAATTTCTGAAT
GGCCCAGGTCTGAGGCCTCCCTTTTTTTTGTCCCCCCAACTTGATGTATGAAGGCTT
TGGTCTCCCTGGGAGGGGGTTGAGGTGTTGAGGCAGCCAGGGCTGGCCTGTACAC
TGACTTGAGACCAATAAAAGTGCACACCTTACCTTACACAAACAGCTTGTGGCTCTGT
GGCTTTGCTGGGTGTGGGGAGCAGGTTGGGTGGGTGTGGAGCTCTATTGGGGGGG
GCATCTAGGGTGGGCTAGGCCTTGCTGATGGTATCTAGTGGGAGGGCT

Actb NHEJ donor (bold: sgRNA target sites)

agtccgcctagaagcacttgcggcgccactaacttctccctgttgaaacaagcaggggatgtcgaagagaatcccggg
ccaatggtgagcaagggcgaggaggataacatggccatcatcaaggagttcatgcgcttcaaggtgcacatggagggct
ccgtgaacggccacgagttcgagatcgagggcgagggcgagggccgcccctacgagggcacccagaccgccaagct
gaaggtgaccaagggtggcccccctgcccttcgcctgggacatcctgtcccctcagttcatgtacggctccaaggcctacgtg
aagcaccccgccgacatccccgactacttgaagctgtccttccccgagggcttcaagtgggagcgcgtgatgaacttcgag
gacggcggcgtggtgaccgtgacccaggactcctccctgcaggacggcgagttcatctacaaggtgaagctgcgcggca
ccaacttcccctccgacggccccgtaatgcagaagaagaccatgggctgggaggcctcctccgagcggatgtaccccga
ggacggcgccctgaagggcgagatcaagcagaggctgaagctgaaggacggcggccactacgacgctgaggtcaag
accacctacaaggccaagaagcccgtgcagctgcccggcgcctacaacgtcaacatcaagttggacatcacctcccaca
acgaggactacaccatcgtggaacagtacgaacgcgccgagggccgccactccaccggcggcatggacgagctgtac
aagtaa**agtccgcctagaagcacttgcgg**

Actb MMEJ donor (bold: sgRNA target sites; upper: micro-homology arm; underline; linker)
agtccgcctagaagcacttgcggCCCCTCCATCGTGCACCGCAAGTGCTTC<u>ttaattaa</u>cgccacta
acttctccctgttgaaacaagcaggggatgtcgaagagaatcccgggccaatggtgagcaagggcgaggaggataacat
ggccatcatcaaggagttcatgcgcttcaaggtgcacatggagggctccgtgaacggccacgagttcgagatcgagggcg
agggcgagggccgcccctacgagggcacccagaccgccaagctgaaggtgaccaagggtggcccccctgcccttcgcct
gggacatcctgtcccctcagttcatgtacggctccaaggcctacgtgaagcaccccgccgacatccccgactacttgaagct
gtccttccccgagggcttcaagtgggagcgcgtgatgaacttcgaggacggcggcgtggtgaccgtgacccaggactcct
ccctgcaggacggcgagttcatctacaaggtgaagctgcgcggcaccaacttcccctccgacggccccgtaatgcagaa
gaagaccatgggctgggaggcctcctccgagcggatgtaccccgaggacggcgccctgaagggcgagatcaagcaga
ggctgaagctgaaggacggcggccactacgacgctgaggtcaagaccacctacaaggccaagaagcccgtgcagctg
cccggcgcctacaacgtcaacatcaagttggacatcacctcccacaacgaggactacaccatcgtggaacagtacgaac
gcgccgagggccgccactccaccggcggcatggacgagctgtacaagtaa<u>ggcgcgcc</u>GCGGACTGTTACTG
AGCTGC**ccgcaagtgcttctaggcggact**

Actb HMEJ donor (bold: sgRNA target sites; upper: homology arm; underline; linker)
agtccgcctagaagcacttgcggCCGGGACCTGACAGACTACCTCATGAAGATCCTGACCGA
GCGTGGCTACAGCTTCACCACCACAGCTGAGAGGGAAATCGTGCGTGACATCAAAG
AGAAGCTGTGCTATGTTGCTCTAGACTTCGAGCAGGAGATGGCCACTGCCGCATCCT
CTTCCTCCCTGGAGAAGAGCTATGAGCTGCCTGACGGCCAGGTCATCACTATTGGCA
ACGAGCGGTTCCGATGCCCTGAGGCTCTTTTCCAGCCTTCCTTCTTGGGTAAGTTGT
AGCCTAGTCCTTTCTCCATCTAAAGGTGACAAAACTCCTGAGGCCATAGTACAAGTTA
AGTCTGATTTCTGTCACTCTTCTCTTAGGTATGGAATCCTGTGGCATCCATGAAACTA
CATTCAATTCCATCATGAAGTGTGACGTTGACATCCGTAAAGACCTCTATGCCAACAC
AGTGCTGTCTGGTGGTACCACCATGTACCCAGGCATTGCTGACAGGATGCAGAAGG
AGATTACTGCTCTGGCTCCTAGCACCATGAAGATCAAGGTAAGCTAAGCATCCTTAGC
TTGGTGAGGGTGGGCCCTGTGGTTGTCAGAGCAACCTTCTAGGTTTAAGGGGAATC
CCAGCACCCAGAGAGCTCACCATTCACCATCTTGTCTTGCTTTCTTCAGATCATTGCT
CCTCCTGAGCGCAAGTACTCTGTGTGGATCGGTGGCTCCATCCTGGCCTCACTGTC
CACCTTCCAGCAGATGTGGATCAGCAAGCAGGAGTACGATGAGTCCGGCCCCTCCA
TCGTGCACCGCAAGTGCTTC<u>ttaattaa</u>cgccactaacttctccctgttgaaacaagcaggggatgtcgaaga
gaatcccgggccaatggtgagcaagggcgaggaggataacatggccatcatcaaggagttcatgcgcttcaaggtgcac

atggagggctccgtgaacggccacgagttcgagatcgagggcgagggcgagggccgcccctacgagggcacccaga
ccgccaagctgaaggtgaccaagggtggccccctgcccttcgcctgggacatcctgtcccctcagttcatgtacggctccaa
ggcctacgtgaagcaccccgccgacatccccgactacttgaagctgtccttccccgagggcttcaagtgggagcgcgtgat
gaacttcgaggacggcggcgtggtgaccgtgacccaggactcctccctgcaggacggcgagttcatctacaaggtgaag
ctgcgcggcaccaacttcccctccgacggccccgtaatgcagaagaagaccatgggctgggaggcctcctccgagcgg
atgtaccccgaggacggcgccctgaagggcgagatcaagcagaggctgaagctgaaggacggcggccactacgacg
ctgaggtcaagaccacctacaaggccaagaagcccgtgcagctgcccggcgcctacaacgtcaacatcaagttggacat
cacctcccacaacgaggactacaccatcgtggaacagtacgaacgcgccgagggccgccactccaccggcggcatgg
acgagctgtacaagtaaggcgcgccGCGGACTGTTACTGAGCTGCGTTTTACACCCTTTCTTTG
ACAAAACCTAACTTGCGCAGAAAAAAAAAAAATAAGAGACAACATTGGCATGGCTTTG
TTTTTTTAAATTTTTTTTAAAGTTTTTTTTTTTTTTTTTTTTTTTTTTTTTAAGTTTTTTTGT
TTTGTTTTGGCGCTTTTGACTCAGGATTTAAAAACTGGAACGGTGAAGGCGACAGCA
GTTGGTTGGAGCAAACATCCCCCAAAGTTCTACAAATGTGGCTGAGGACTTTGTACA
TTGTTTTGTTTTTTTTTTTTTTTGGTTTTGTCTTTTTTTTAATAGTCATTCCAAGTATCCAT
GAAATAAGTGGTTACAGGAAGTCCCTCACCCTCCCAAAAGCCACCCCCACTCCTAAG
AGGAGGATGGTCGCGTCCATGCCCTGAGTCCACCCCGGGGAAGGTGACAGCATTG
CTTCTGTGTAAATTATGTACTGCAAAAATTTTTTAAATCTTCCGCCTTAATACTTCATTT
TTGTTTTTAATTTCTGAATGGCCCAGGTCTGAGGCCTCCCTTTTTTTTGTCCCCCCAA
CTTGATGTATGAAGGCTTTGGTCTCCCTGGGAGGGGGTTGAGGTGTTGAGGCAGCC
AGGGCTGGCCTGTACACTGACTTGAGACCAATAAAAGTGCACACCTTACCTTACACA
AACAGCTTGTGGCTCTGTGGCTTTGCTGGGTGTGGGGAGCAGGTTGGGTGGGTGT
GGAGCTCTATTGGGGGGGGCATCTAGGGTGGGCTAGGCCTTGCTGATGGTATCTAG
TGGGAGGGCT**ccgcaagtgcttctaggcggact**
